# Evaluating Encoder and Decoder Models for Extended Clinical Concept Recognition in Japanese Clinical Texts: Comparative Study With Weighted Soft Matching

**DOI:** 10.2196/78681

**Published:** 2026-05-14

**Authors:** Yuya Tsukiji, Satoshi Kataoka, Masafumi Itokazu, Ryozo Nagai, Takeshi Imai

**Affiliations:** 1 Center for Disease Biology and Integrative Medicine, Graduate School of Medicine, The University of Tokyo, 7-3-1 Hongo, Bunkyo-ku, Clinical Research Center A646, The University of Tokyo Hospital, Tokyo, 1138655, Japan, 81 03-5841-3454; 2 Jichi Medical University, Tochigi, Japan

**Keywords:** natural language processing, NLP, named entity recognition, instruction tuning, token classification, large language model, LLM, transformer model

## Abstract

**Background:**

Extracting medical knowledge for secondary purposes, such as diagnostic support, continues to pose a substantial challenge. Conventional named entity recognition has focused on short terms (eg, genes, diseases, and chemicals), whereas extraction and assessment of longer, complex expressions remain underexplored. Clinically vital concepts, such as diseases, pathologies, symptoms, and findings, often appear as long phrases, and accurate extraction is crucial for applications such as constructing causal knowledge from case reports. Consequently, a framework addressing both short terms and clinically meaningful long phrases—termed extended Clinical Concept Recognition (E-CCR)—is essential.

**Objective:**

This study, the first comprehensive investigation of E-CCR model selection, aimed to identify optimal strategies by comparing encoder versus decoder models and general-purpose versus domain-specific pretraining. We analyzed variation in effectiveness by target length and proposed a novel E-CCR evaluation metric.

**Methods:**

We evaluated 17 encoder and decoder models using J-CaseMap, a database of approximately 20,000 Japanese case reports annotated with clinical concepts. Performance was primarily assessed using the weighted soft matching score, which penalizes fragmentation of long extraction targets and weights scores by target length to account for the greater difficulty of extracting longer expressions.

**Results:**

On J-CaseMap, JMedDeBERTa(s)—an encoder model pretrained on domain-specific medical text—achieved the highest mean performance (F1-score=0.758, SD 0.002), with similarly strong results from JMedDeBERTa(c), suggesting comparable performance among the top encoder models. As the fragmentation penalty increased, performance generally declined; however, no consistently severe degradation was observed. On the Medical Report Named Entity Recognition for positive disease dataset, the general-domain DeBERTaV2-base yielded the highest mean F1 score, and differences among the medical-domain JMedDeBERTa(s) and JMedDeBERTa(c) variants were small, suggesting limited benefit of domain-specific pretraining. Overall, under our experimental settings (low-rank adaptation fine-tuning for decoders and full fine-tuning for encoders), encoder models outperformed decoder models, and token classification outperformed our instruction tuning setup.

**Conclusions:**

Under our experimental setting, encoder-based token classification achieved the highest mean performance on our internal dataset. Differences among the top encoder models were small and should be interpreted as comparable within the uncertainty implied by our annotation review, whereas decoder-based approaches did not surpass encoder-based models in this setup, suggesting that encoder models can deliver high accuracy with fewer parameters and may offer practical advantages in resource-constrained environments. Token classification outperformed instruction tuning for extracting long expressions, whereas instruction tuning was better suited to short terms. Using the weighted soft matching score, we found that performance did not substantially deteriorate as the fragmentation penalty increased, indicating that extracted spans were rarely fragmented. Similar trends in external validation datasets suggest that findings under our setup may generalize to information extraction tasks on Japanese medical text. Further investigation is needed to determine whether these findings hold across other languages and medical document types.

## Introduction

With advancements in digitization within the medical field, various medical documents, including electronic medical records, case reports, and discharge summaries, are increasingly digitized. There is a growing demand for the secondary use of these digitized documents, such as extracting medical knowledge for diagnostic support, and research in this area is advancing. For example, Japan has developed a diagnostic support system called “Difficult Diagnosis Case Search: J-CaseMap” [1]. J-CaseMap uses a manually constructed knowledge graph created by approximately 150 internists, extracting the causal relationships between diseases and the associated pathophysiological processes and symptoms from approximately 20,000 case reports collected from local chapters of the Japan Society of Internal Medicine. A system has been developed that, through search and inference on this large-scale knowledge graph integrating all causal chains from these cases, presents both a “list of differential diagnoses suggested by a given combination of input symptoms and findings” and “the causal chain leading to those diagnoses.” This system has been implemented as a service for members of the Japan Society of Internal Medicine.

Such diagnostic support systems can assist in searching similar cases, suggesting diseases, and supporting diagnostic reasoning, making them highly valuable for standardizing the quality of medical care. However, constructing and updating the underlying structured knowledge database requires substantial time and effort, posing substantial challenges to efficiency and automation. Consequently, demand for automated methods leveraging large language models (LLMs), which have seen rapid advances in recent years, has increased to structure knowledge from medical documents using computers.

When constructing a knowledge database of the causal relationships among diseases, pathologies, symptoms, and findings that are considered essential for differential diagnosis from clinical texts (such as case reports), the first crucial step is to extract the terms and phrases representing these key clinical concepts. Conventionally, named entity recognition (NER) has been widely used to extract patient-related information from medical texts, including clinical records, medical literature, and electronic health records [2,3]. The expressions extracted can then be used for aggregating clinical data, substantiating biomedical findings, supporting clinical decision-making, improving patient care, and enhancing health care management and resource allocation [4]. However, much of the prior research has concentrated solely on extracting terms or short expressions (eg, disease names, symptoms, drug names, and treatment methods) [5-10].

The clinical concepts critical for differential diagnosis—diseases, pathologies, symptoms, and findings—are not necessarily confined to single terms or short expressions. For instance, the expression “Anti-GAD antibody level was elevated at 3790.0 U/mL” in its entirety denotes the concept “anti-GAD-positive.” If this concept is considered as a node in a causal knowledge graph, then extracting the corresponding long expression from the text becomes essential. Thus, when addressing the task of “extracting medical concepts that reflect the clinically meaningful units considered by physicians during diagnosis,” a comprehensive extraction framework is needed that targets not only terms (short expressions) but also longer expressions (long expressions) that may span multiple sentences. In this study, this task is referred to as Extended Clinical Concept Recognition (E-CCR). This distinguishes our task from conventional NER, which typically targets shorter named entities such as disease names, drug names, or laboratory items.

Several studies [11-13] have investigated Japanese medical text processing, including work on deidentification, document classification, and extraction of relatively short medical entities. However, to our knowledge, no prior study has systematically addressed the extraction of “clinically important long expressions” that form the nodes of a clinical knowledge graph.

Previous studies have primarily used evaluation metrics such as ROUGE-1, ROUGE-2, ROUGE-L, recall, precision, and *F*_1_-score. ROUGE calculates scores based on string similarity [14]. *F*_1_-scores have been computed by segmenting annotation schemes (ie, the extraction targets) into tokens [2] or characters [15]. Scores are typically computed on a token or character basis rather than per extracted unit, as the number of characters in each extraction target may vary. However, when comparing multiple models, token-based metrics should be avoided because each LLM uses its tokenizer and varying tokenizers yield different segmentation granularities. Conversely, even when ROUGE or *F*_1_-scores are computed on a character basis, their validity is questionable for long extraction targets. This is because a fragmented extraction of a long expression, although undesirable, may still attain a high score on a character basis, simply because the majority of characters are captured. Therefore, when comparing multiple models that include long expressions as extraction targets, it becomes imperative to develop a new evaluation metric that penalizes fragmented outputs and is unaffected by differences in tokenizers.

Expression extraction can be approached as either a sequence labeling task or a generation task. Conventionally, many researchers have addressed expression extraction as a sequence labeling task. Techniques such as conditional random fields [16] and long short-term memory (LSTM) [17] have been proposed, with transformer-based encoder models, particularly those based on Bidirectional Encoder Representations from Transformers (BERT) [18], gaining substantial popularity [19]. More recently, with the emergence of LLMs, interest in performing expression extraction as a generation task has increased, and various information extraction techniques such as cross-domain learning, zero-shot prompting, in-context learning, supervised fine-tuning, and data augmentation have emerged [20]. However, as generation tasks require simultaneous text generation and expression extraction, they are inherently more complex than sequence labeling approaches. Therefore, extracting expressions via generation tasks necessitates careful prompt design and fine-tuning [21-25].

However, whether encoder or decoder models are more suitable for expression extraction remains unclear. For example, even when techniques such as designing prompt formats that facilitate the proper noun extraction [22], tailoring prompts to specific tasks [23], and customizing datasets [24] are used, in zero-shot or few-shot settings, decoder models (eg, GPT-3 [25], GPT-3.5, and GPT-4 [26]) have not outperformed encoder models (eg, BERT [23], BioClinicalBERT [24], and RoBERTa-Large [27]). In contrast, BioNER-LLAMA, a decoder model specialized for the medical domain, has reportedly surpassed the encoder model BioBERT by using instruction tuning on a dataset for proper noun extraction in biomedical contexts and by using carefully designed prompts [28]. In another study [29], when encoder and decoder models were both used for token classification in expression extraction, LLaMA2 outperformed RoBERTa.

Additionally, insights into the effectiveness of domain-specific texts for expression extraction in the medical domain remain limited. Previous studies have compared models pretrained on domain-specific medical texts or further adapted to them with models pretrained on general text. For example, BERT_mimic, pretrained on medical texts, demonstrated superior performance compared to a BERT model pretrained on general English texts [30]. Similarly, BioBERT, initially pretrained on general English texts and subsequently adapted to medical texts, outperformed BERT [31]. In contrast, BioELECTRA, pretrained on a relatively small corpus (0.8 GB) of German medical texts, performed worse than DBMDZ ELECTRA, which was pretrained on general texts. Lentzen et al [32] reported that BioGottBERT—first pretrained on general texts and then further pretrained on similar medical texts—achieved the best performance in some cases. Subies et al [33] confirmed the effectiveness of encoder models in Spanish clinical tasks but noted that the best-performing model was not domain adapted; rather, domain-adapted models performed worse than general or multilingual models. Thus, the current insights regarding the effectiveness of domain-adapted models remain inadequate. Moreover, prior studies have primarily focused on extracting short expressions such as drug names, disease names, symptoms, and treatment methods, and evidence on long expression extraction remains limited.

Therefore, this study aimed to elucidate effective model selection strategies for the E-CCR task by comparing encoder and decoder models, as well as general-purpose models with domain-specific models. We propose a novel evaluation metric tailored for E-CCR that not only incorporates conventional measures but also accounts for penalties when long extraction targets are fragmented. We analyzed how model effectiveness varies with the length of the extraction target.

## Methods

### Overview

**Figure 1.**
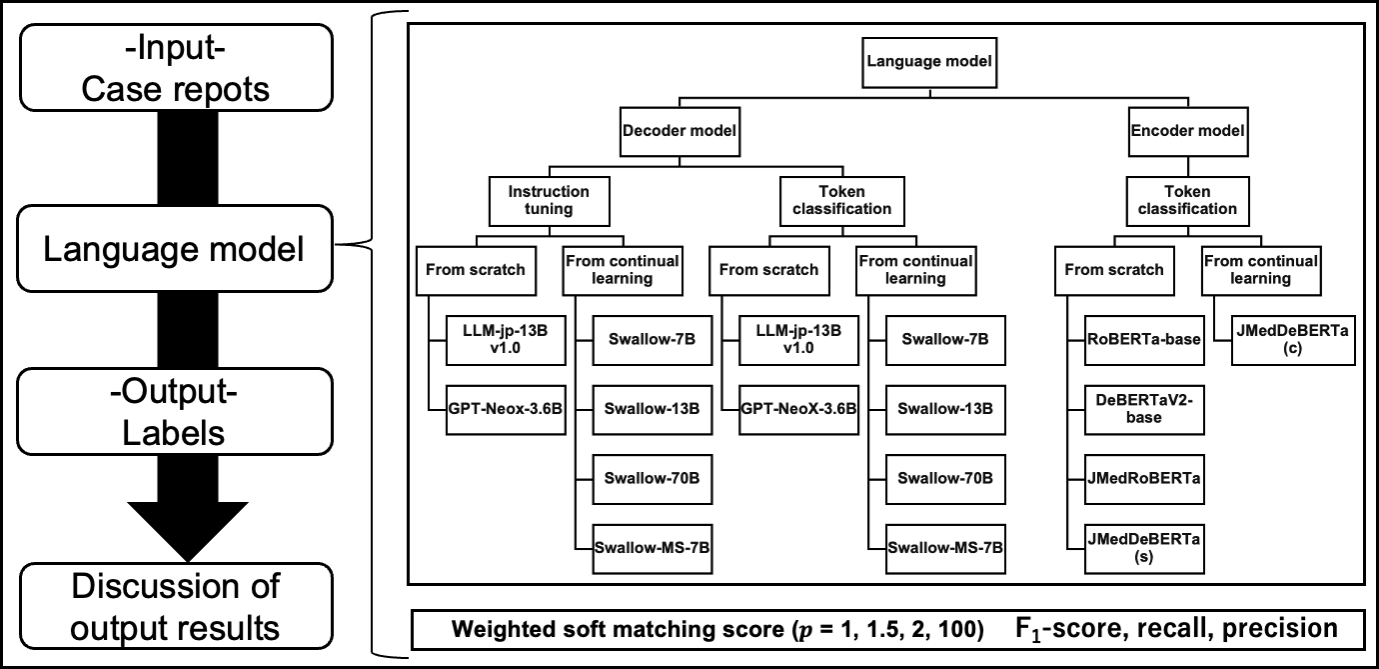
Overall study design for comparing encoder and decoder language models on the extended Clinical Concept Recognition task. Japanese clinical case reports from the J-CaseMap database were used as input texts. Seventeen language models (encoder and decoder architectures and instruction tuning vs token classification settings) were fine-tuned in a 5-fold cross-validation framework to extract clinically important concepts from these reports. Model outputs were evaluated using the weighted soft matching score with varying fragmentation penalties (*p*=1, 1.5, 2, 100), along with conventional *F*_1_, recall, and precision, followed by qualitative review of output examples.

Figure 1 illustrates the flowchart of our research design. We compared and evaluated sequence labeling tasks (token classification) with generation tasks (instruction tuning) using a corpus of clinically essential concepts extracted from case reports. Performance was assessed using a weighted soft matching score, and 17 measures—including *F*_1_-score, recall, and precision—were used for both encoder and decoder models.

### Materials

We used data from the J-CaseMap database, which comprises abstracts from approximately 20,000 case reports presented at local chapters of the Japanese Society of Internal Medicine. Each abstract contains approximately 600 characters. The database was manually constructed by approximately 150 internists, who extracted causal chains of diseases, resulting pathophysiological processes, and associated clinical signs and symptoms from these case reports. The knowledge graph comprises “nodes,” representing term expressions that are normalized by specific rules, and “edges,” which represent the causal relationships between them. Although such a knowledge database supports decision systems in differential diagnosis, its construction requires substantial manual effort, thereby necessitating the development of automated methods.

Using J-CaseMap as an example, the task of automatically constructing a causal chain knowledge base from case reports can be divided into three steps:

1. Extraction: identify and extract terms or phrases from the case reports that are considered clinically essential for differential diagnosis (eg, diseases, pathophysiological processes, signs, and symptoms)
2. Normalization: map the extracted expressions to standardized terminology
3. Causal inference: infer causal relationships among the normalized expressions

**Figure 2.**
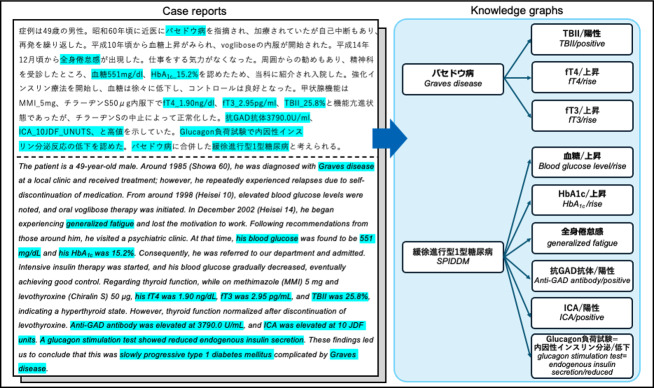
Correspondence between clinically essential expressions in a Japanese case report and the J-CaseMap knowledge graph. The left panel shows an example Japanese case report (with English translation) describing type 1 diabetes complicated by Graves disease. Phrases highlighted in light blue indicate text spans selected by physicians as clinical concepts (eg, diagnoses, symptoms, and test findings) that are important for differential diagnosis. The right panel illustrates how these expressions are normalized as nodes and linked by causal relationships (edges) within the J-CaseMap knowledge graph. These node-defining spans constitute the gold-standard corpus for the extended Clinical Concept Recognition task. GAD: glutamic acid decarboxylase; HbA_1c_: hemoglobin A_1c_; ICA: islet cell antibody; SPIDDM: slowly progressive insulin-dependent diabetes mellitus; TBII: thyrotropin binding inhibitory immunoglobulin.

As shown in Figure 2, the first critical step in developing an automated approach is extracting clinically essential concepts that form the nodes of the knowledge graph. When these essential clinical concepts appear in the text, they often manifest as long expressions. For instance, the sentence “Glucagon負荷試験で内因性インスリン分泌反応の低下を認めた” might be represented as “Glucagon負荷試験 = 内因性インスリン分泌/低下” (ie, *“The glucagon stimulation test indicated a reduced endogenous insulin secretory response” → “glucagon stimulation test=endogenous insulin secretion/reduced”*), constituting a long expression.

Using the data from J-CaseMap, we created a corpus of approximately 150,000 pairs of sentences. Six nurses identified segments corresponding to the normalized “term expressions” manually from the original case report sentences. This corpus was used to define clinically essential expressions in sentences as descriptions and definitions of nodes in the knowledge graph of J-CaseMap. This study aimed to extract these clinically meaningful expressions in sentences. Converting expressions from extracted sentences into normalized terminology was beyond the scope of this study.

The medical text corpus was used to fine-tune the encoder and decoder models. We used 0.56 GB of medical domain texts, which combined real-world clinical texts, such as discharge summaries (0.53 GB), and textbook-style medical texts, such as medical textbooks (0.03 GB), to construct a unique DeBERTa [34] pretraining model and an additional pretraining model with complex medical knowledge.

### Annotation Procedure

To minimize the subjectivity inherent in the definition of “clinically important expressions,” we designed our annotation scheme on the basis of the J-CaseMap knowledge graph. For each case report, we first regarded as “descriptions important for clinical reasoning” those concepts that the physicians editing J-CaseMap had adopted as component nodes of the knowledge graph for that case (this node selection process itself involved multiple rounds of review by several physicians). We then constructed the corpus by marking, in the original case report text, the descriptions that corresponded to these node expressions. The annotation task was performed by 6 nurses, with 2 nurses independently annotating each case report. When their results did not agree, the 2 annotators discussed the discrepancies and revised the annotations until full consensus was reached. However, because we did not retain the preadjudication annotations, we could not compute the initial interannotator agreement (IAA). Accordingly, the resulting labels should be interpreted as an adjudicated consensus reference standard rather than an objective ground truth. Thus, our evaluation primarily quantifies agreement with this consensus, and some apparent “errors” may reflect clinically acceptable alternative span selections rather than model failures.

### Creating Correct Answer Label Data

**Figure 3.**
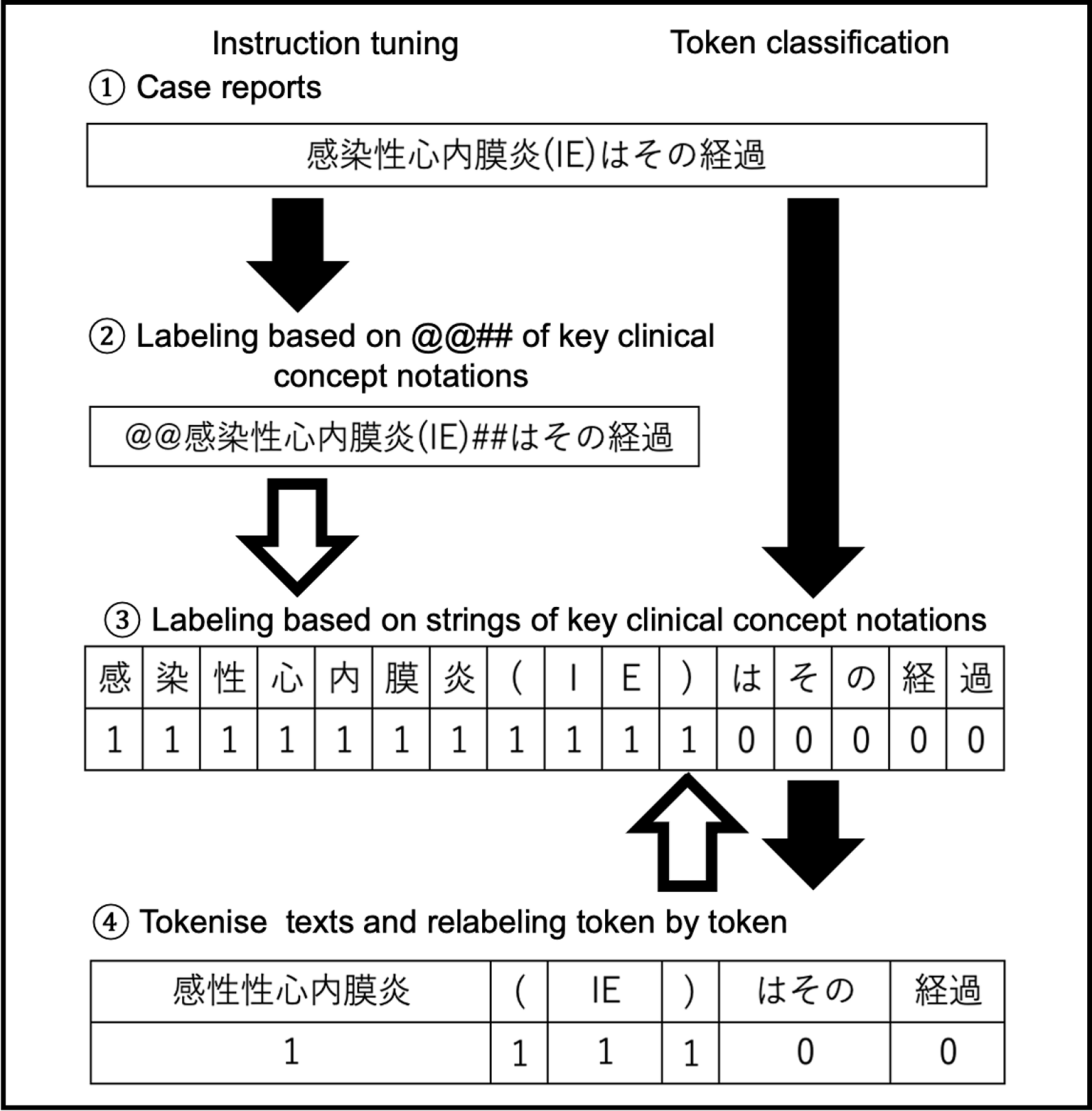
Creation of gold-standard labels and model inputs for instruction tuning versus token classification. The example sentence “感染性心内膜炎 (IE）はその経過” (“The course of infective endocarditis (IE)”) is taken from a Japanese clinical case report. In the instruction tuning setting (left), clinically important concepts within the sentence are enclosed by special markers (@@ and ##) in the output text. In the token classification setting (right), the same sentence is split into characters, and binary labels are assigned to each character (1 for characters within clinically essential expressions and 0 otherwise), which are then mapped to tokens based on the tokenization. Character-level labels are used to calculate the weighted soft matching score, enabling fair comparison across models with different tokenizers.

Figure 3 illustrates the process for generating correct label data and handling input and output for the model.

In instruction tuning, following Wang et al [23] and Keloth et al [28], ① case report sentences are provided as input to the models, and ② important clinical concepts are labeled by enclosing them with @@ and ## markers. These marked expressions are then extracted, and performance evaluation is conducted after splitting the text into characters (③). We enabled byte fallback to ensure robust handling of out-of-vocabulary characters.

In token classification, ① case report sentences are split into characters (③); a label of “1” is assigned to character strings identified as critical clinical concepts, and “0” is assigned to all other character strings. The case report text is then tokenized and labeled with “1” for tokens representing important clinical concepts and “0” for all other tokens. At this point, if the byte fallback function of each model is enabled, expressions that are not in the vocabulary set of the tokenizer are subdivided, making it difficult to label them (eg, “蛔⾍(*Ascaris*)” → “‘<0xE8>’, ‘<0x9B>’, ‘<0x94>’, ‘虫’”). Notably, the encoder models used in this study originally had byte fallback disabled by default. Accordingly, for consistency across models and to avoid label fragmentation, the byte fallback function was disabled in all encoder and decoder models used for token classification, and unknown tokens were allowed. Next, the case report sentences are input to multiple models, and the labels after step ④ represent the model outputs. Performance evaluation is conducted on a character-by-character basis.

Performance was evaluated on the labels generated after the aforementioned steps for both tasks. The performance evaluation used a weighted soft matching score inspired by the Hölder mean.


$${\left(\sum_{i=1}^{m}{\left(\frac{a_{i}}{l}\right)}^{p}\right)}^{\frac{1}{p}}$$

For each extracted entity, *l* is the length of the entity string, *m* is the number of markers assigned to that entity, and *a*_*i*_ is the string length of the *i*-th marker. Finally, *p* represents the penalty strength.

This evaluation metric imposes a penalty on the extraction process when segmented parts are present. Each extraction target was scored using this weighted soft matching score; furthermore, considering that extracting longer targets is more challenging, the scores were aggregated using a length-weighted average based on the target length. This assigns relatively higher scores to more challenging extractions. This evaluation metric is designed such that when *p*=1, it corresponds to a character-level evaluation. As *p* increases, there is a penalty for the extracted part if there is a split in the extracted marker, and when *p=∞*, it converges to the ratio of the longest extracted part in the extracted target.

Performance was also assessed on a marker-by-marker basis (marker matching score)—a simple average that does not account for target length—as well as on token-split and character-split bases.

Results are presented in Tables S1-S3 in Multimedia Appendix 1. In this study, the number of extraction targets differed for each case; therefore, we evaluated all target extraction markers contained in multiple instances in the test set. Case-specific performance evaluations are presented in Tables S4-S7 in Multimedia Appendix 1.

Using the aforementioned evaluation metrics, we computed true positives, false positives, and false negatives, focusing on the target extraction spans, and then calculated the *F*_1_-score from these values. We did not count true negatives for strings outside the extraction targets; therefore, even if instruction tuning produces outputs that differ from the source text in nonextraction spans, such differences do not affect the evaluation.

### Training Settings

Token classification and instruction tuning were conducted on the corpus of important clinical concepts using the following models:

- General-purpose models pretrained on Japanese text include:
  - RoBERTa [35] (rinna/japanese-roberta-base; hereinafter RoBERTa-base)
  - DeBERTa (izumi-lab/deberta-v2-base-japanese; hereinafter DeBERTaV2-base)
  - GPT2 [36] (llm-jp/llm-jp-13b-v1.0, llm-jp/llm-jp-13binstruct-full dolly_en-dolly_ja-ichikara_003_001-oasst_en-oasst_ja-v1.1; hereinafter LLM-jp-13B v1.0)
  - GPT-NeoX [37] (rinna/japanese-gpt-neox-3.6b, rinna/ japanese-gpt-neox-3.6b-instruction-sft-v2; hereinafter GPT-NeoX-3.6B)
- Domain-specific models pretrained on medical texts include:
  - RoBERTa (alabnii/jmedroberta-base-sentencepiecevocab50000; hereinafter JMedRoBERTa)
  - DeBERTa pretrained on original medical texts (JMedDeBERTa(s))
- Models additionally pretrained on general-purpose Japanese text include:
  - LLaMA2 [38]: (tokyotech-llm/Swallow-7bhf, tokyotech-llm/Swallow-7b-instruct-v0.1, tokyotech-llm/Swallow-13b-hf, tokyotech-llm/ Swallow-13b-instruct-v0.1, tokyotech-llm/Swallow-70b-hf, tokyotech-llm/Swallow-70b-instruct-v0.1, hereinafter Swallow-7B, Swallow-13B, and Swallow-70B)
  - Mistral [39]: (tokyotech-llm/Swallow-MS-7b-v0.1, tokyotech-llm/Swallow-MS-7b-instruct-v0.1, hereinafter Swallow-MS-7B)
- Models further pretrained on medical texts include:
  - DeBERTa (izumi-lab/deberta-v2-base-japanese) with additional pretraining on medical texts (hereinafter JMedDeBERTa(c))

In the list mentioned earlier, the descriptions in parentheses represent the model identifiers on Hugging Face, the largest data-sharing platform for LLMs.

Prior research has reported that even slight modifications in prompt design can markedly alter model performance, indicating that developing effective prompts requires domain-specific expertise [2]. On the basis of the studies by Wang et al [23] and Keloth et al [28], we designed the prompt for instruction tuning as follows: “以下は, タスクを説明する指示と, 文脈のある入力の組み合わせです。” “要求を適切に満たす応答を書きなさい。\n\n” “### 指示:\n与えられた文に対して重要臨床概念を@@と##で強調表示し, 抽出してください。” “重要臨床概念が存在しない場合は同じ文を出力してください。\n\n### 入力:{input}\n\n### 応答:” (*“The following is a combination of instructions describing a task and contextual input.” “Write a response that appropriately fulfills the request.” “###Instruction:\n For the given sentence, highlight the key clinical concepts using @@ and ##, and extract them.” “If there are no key clinical concepts, output the same sentence as it is. \n\n### Input: {input}\n\n### Response:”*) After fine-tuning, we compared these 17 models.

### Constructing the Pretraining and Additional Pretraining Models Using Medical Texts

**Figure 4.**
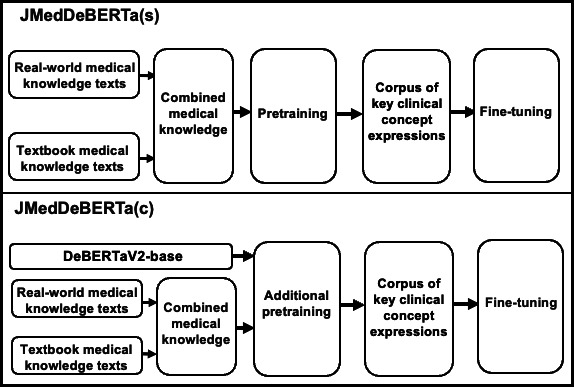
Construction of JMedDeBERTa(s) and JMedDeBERTa(c) using Japanese medical texts. JMedDeBERTa(s) is an encoder model with the DeBERTaV2-base architecture pretrained from scratch on 0.56 GB of Japanese medical text (0.53 GB clinical documents such as discharge summaries and 0.03 GB medical textbooks), then fine-tuned on the extended Clinical Concept Recognition (E-CCR) data set derived from J-CaseMap. JMedDeBERTa(c) starts from a general-domain DeBERTaV2-base model pretrained on 362 GB of mixed-domain Japanese text, continues pretraining on the same 0.56 GB medical corpus, and is then fine-tuned on the same E-CCR data set.

As shown in Figure 4, 0.56 GB of medical domain text was used to construct JMedDeBERTa(s) and JMedDeBERTa(c). When constructing JMedDeBERTa(s), SentencePiece [40] was used as the tokenizer.

For JMedDeBERTa(s) and JMedDeBERTa(c), pretraining and additional pretraining were conducted on 8 V100 GPUs (16 GB each). The medical corpus was split into training and validation sets at a ratio of 0.8:0.2, and the models were trained using the hyperparameters listed in Table S8 in Multimedia Appendix 1. The effective global batch size was 2048 sequences (32 per GPU×8 GPUs, with gradient accumulation of 8). Under this setting, the maximum of 60 training epochs corresponded to 81,960 optimizer update steps (1366 updates per epoch). Therefore, the learning curves in Figures S1 and S2 in Multimedia Appendix 1 represent the full 60-epoch training run, plotted up to approximately 80,000 update steps. In general, substantially larger datasets are required to learn robust language representations. As our domain corpus is relatively small, we additionally monitored the masked language modeling (MLM) loss for both JMedDeBERTa(s) and JMedDeBERTa(c) to verify that training proceeded appropriately.

### Fine-Tuning

#### Overview

**Figure 5.**
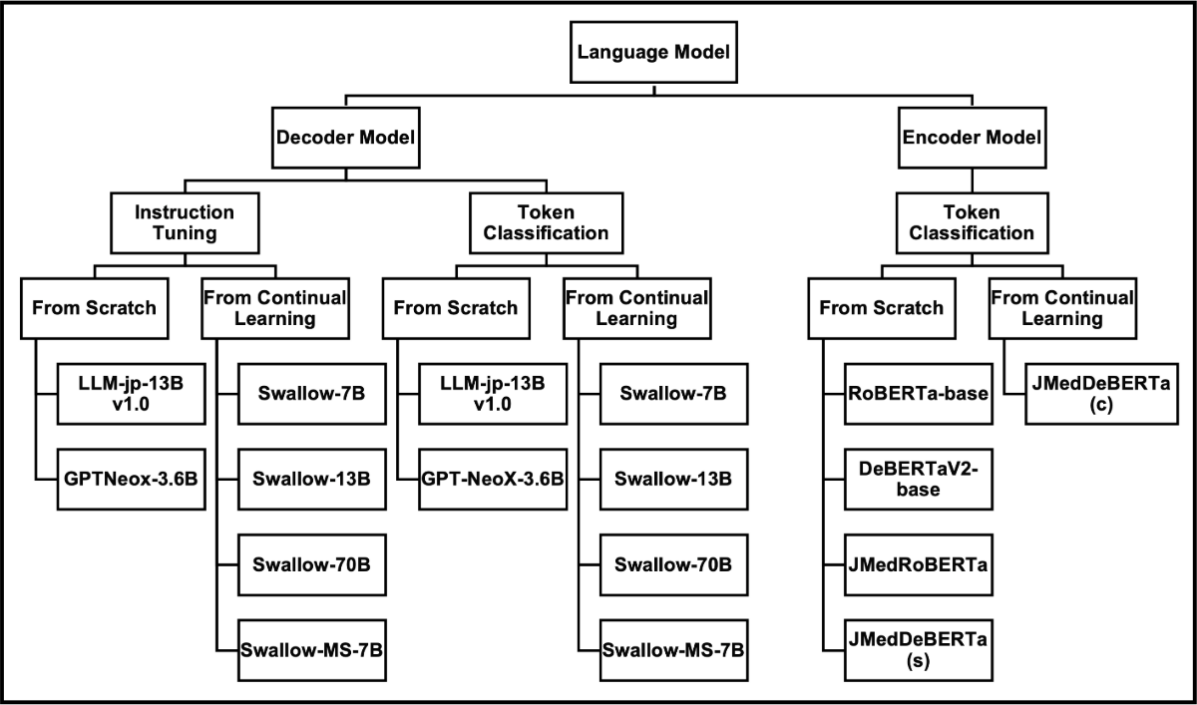
Overview of the 17 encoder and decoder language models compared in this study. All models were fine-tuned to extract clinically important concepts from Japanese case reports in the J-CaseMap database and evaluated on the E-CCR task. Decoder models (LLM-jp-13B v1.0, GPT-NeoX-3.6B, Swallow-7B/13B/70B, Swallow-MS-7B) were fine-tuned by instruction tuning or token classification, while encoder models (RoBERTa-base, DeBERTaV2-base, JMedRoBERTa, JMedDeBERTa(s), JMedDeBERTa(c)) were fine-tuned by token classification only. All models used identical data splits and evaluation metrics.

Figure 5 lists the 17 comparison models used for fine-tuning, and Table 1 provides an overview of the various models. For fine-tuning, the corpus of clinically significant concepts was divided into training, validation, and test sets at a ratio of 0.6:0.2:0.2, and 5-fold cross-validation was performed for 10 epochs (with early stopping). The 70B decoder model could be trained on an NVIDIA H100 (80 GB) GPU, while all other models were trainable on a single NVIDIA A6000 (48 GB) GPU. The hyperparameters for the encoder and decoder models were selected based on settings used in prior studies on NER tasks [28,29] and are detailed in Table S9 in Multimedia Appendix 1. Fine-tuning performance metrics included F1 score, recall, and precision, evaluated using the weighted soft matching score. Due to computational resource constraints, we applied low-rank adaptation (LoRA) to the decoder models and full fine-tuning to the encoder models. This choice was motivated by prior work showing that LoRA can achieve performance comparable to full fine-tuning [41].

**Table 1.** Characteristics of the 17 encoder and decoder language models evaluated on the extended clinical concept recognition task in Japanese clinical case reports.

Models^a^	Architecture	Parameters	Pretraining methods	Pretraining corpus type	Pretraining corpus size	Vocab size
Decoder models						
GPT-NeoX-3.6B	GPT-NeoX-3.6B	3.6B	From scratch	General (Japanese)	312.5B tokens	32,000
LLM-jp-13B v1.0	GPT2-13B	13B	From scratch	General (Japanese)	300B tokens	50,570
Swallow-7B	LLaMA2-7B	7B	Continual learning	English+general (Japanese)	+100B tokens	43,176
Swallow-13B	LLaMA2-13B	13B	Continual learning	English+general (Japanese)	+100B tokens	43,176
Swallow-70B	LLaMA2-70B	70B	Continual learning	English+general (Japanese)	+100B tokens	43,176
Swallow-MS-7B	Mistral-7B	7B	Continual learning	English+general (Japanese)	+105B tokens	42,800
Encoder models						
DeBERTaV2-base	DeBERTa V2-base	110M	Continual learning	General+finance	357 GB+5.2 GB	32,000
RoBERTa-base	RoBERTa-base	110M	From scratch	General (Japanese)	75 GB	32,000
JMedRoBERTa	RoBERTa-base	124M	From scratch	Medical (Japanese)	1.8 GB	50,000
JMedDeBERTa(s)	DeBERTa V2-base	125M	From scratch	Medical (Japanese, this study)	0.56 GB	32,000
JMedDeBERTa(c)	DeBERTa V2- base	110M	Continual learning	General+medical (this study)	362 GB (general)+0.56 GB (medical)	32,000

a Models are grouped by architecture (decoder or encoder) and described by parameter count, pretraining methods, pretraining corpus type and size, and vocabulary size. All models were subsequently fine-tuned on the data set constructed from the J-CaseMap database.

For decoder-only models in the token classification setting, we retained the default causal (left-to-right) self-attention behavior used during pretraining; thus, the hidden state at position t could attend only to tokens at positions ≤t. In implementation, we used only the standard padding attention mask to ignore padded tokens and did not modify the causal mask or the attention pattern (eg, no full unmasking, no layer-wise causal mask removal, and no other bidirectionality-enabling modification) [42,43]. We attached a token-level classification head to the final-layer hidden states and computed label logits for all token positions in parallel from a single forward pass under this causal mask.

#### Pretraining Methods

“From scratch” indicates that the base model was pretrained from random initialization on the corpus listed under “pretraining corpus type/size.” “Continual learning” indicates that the base model was initialized from an existing pretrained checkpoint and further pretrained on additional corpora (eg, Swallow models continued training on approximately 100 billion tokens of English plus general Japanese text following LLaMA2 or Mistral; JMedDeBERTa(c) continued training on 0.56 GB of medical text after DeBERTaV2-base).

#### Pretraining Corpus Type

“General” indicates general-domain Japanese text; “medical” indicates the 0.56 GB of Japanese medical documents used in this study; “general+finance” indicates a mixture of general and financial text; and “general+medical” indicates the combination of the general corpus used for DeBERTaV2-base and the 0.56 GB medical corpus used for JMedDeBERTa(c).

### External Evaluation on a Public Clinical NER Dataset

To assess the reproducibility and generalizability of our findings using a public benchmark, we conducted an additional evaluation on Medical Report Named Entity Recognition for positive disease (MRNER disease), a Japanese clinical NER dataset used in JmedBench [44]. MRNER disease comprises 100 clinical documents (50 case reports and 50 radiology reports) and 810 annotated entities. The task focuses on extracting relatively short disease names actually observed in patients, corresponding to a conventional clinical NER setting rather than our E-CCR task, which targets longer, diagnostically important phrases.

Although the number of annotated entities is relatively small for training large models, we considered the dataset sufficient for benchmark evaluation. We applied the same preprocessing, fine-tuning procedures, and evaluation framework as in the main analysis. Specifically, all 17 models were fine-tuned on MRNER disease and evaluated using the weighted soft matching score with fragmentation penalty parameter *p*=1, 1.5, 2, 100. Detailed scores for each model (*F*_1_-score, recall, and precision at each *p*) are reported in Tables S10-S17 in Multimedia Appendix 1.

### Ethical Considerations

In this study, to protect patient privacy, all personally identifiable information was removed from the datasets used. The case reports of the Japanese Society of Internal Medicine used to construct the J-CaseMap database are abstracts of case reports presented at regional meetings of the Society that do not contain personal information, and they have already been made publicly available on the web; therefore, no additional ethical review was required. In contrast, for the development of the language model using clinical record texts, to protect patient privacy, we used data from which all traceable personally identifiable information had been removed from the analysis dataset, and the study was conducted in accordance with the Declaration of Helsinki with the approval of the Ethics Committee of the Faculty of Medicine and Graduate School of Medicine, The University of Tokyo (approval 2018-NI). As this study analyzed only anonymized case report texts (secondary-use data) and involved no direct intervention with or interaction toward human subjects, the ethics committee determined that neither individual informed consent from patients nor financial compensation was required. Neither this article nor the supplementary materials contains any images or other materials that could be used to identify individual patients or clinicians.

## Results

### Overview

Table 2 summarizes the average results from 5-fold cross-validation of the 17 comparison models. Tables S1-S3 in Multimedia Appendix 1 display the average results for markers, characters, and tokens across all cases. Tables S4-S7 in Multimedia Appendix 1 show average results for weighted soft matching scores, marker matching scores, characters, and tokens for each case. Model performance declined as the penalty for splits in extracted parts increased. When comparing the results for *p=1* and *p*=100, the decoder model GPT-NeoX-3.6B was impacted, with an *F*_1_-score reduction of −0.047.

For other decoder models using token classification, the *F*_1_-score ranged from −0.042 to −0.046. Encoder model *F*_1_-scores ranged from −0.032 to −0.036. When using instruction tuning, the *F*_1_-score for all decoder models ranged from −0.022 to −0.030.

Regardless of the penalty size, the encoder model using token classification, that is, JMedDeBERTa(s), achieved the highest mean scores, while the top encoder models showed very similar performance. The decoder model using instruction tuning, that is, GPT-NeoX-3.6B, showed the worst results. Among the decoder models, Swallow-70B, which exhibited the best results for instruction tuning and token classification, could not produce better results than the encoder models.

Next, we compared and verified the results for *p*=1, which performed the best. Among all the models, the encoder model using token classification, that is, JMedDeBERTa(s), achieved the highest performance: *mean F*_1_-score 0.758 (SD 0.002), mean recall 0.768 (SD 0.011), and mean precision 0.749 (SD 0.009). In contrast, the decoder model GPT-NeoX-3.6B, under instruction tuning, recorded the lowest performance: mean *F*_1_-score 0.621 (SD 0.023), mean recall 0.582 (SD 0.044), and mean precision 0.669 (SD 0.006). The encoder model JMedDeBERTa(s), with 125 million parameters, is approximately 1/70th the size of Swallow-70B with 70 billion parameters. The decoder model Swallow-70B, under instruction tuning, achieved mean *F*_1_-score 0.713 (SD 0.016), mean recall 0.680 (SD 0.035), and mean precision 0.751 (SD 0.020). The decoder model using token classification, that is, Swallow-70B, achieved mean *F*_1_-score 0.739 (SD 0.005), mean recall 0.744 (SD 0.013), and precision mean 0.734 (SD 0.003). The metric scores of JMedDeBERTa(s) were better than those of Swallow-70B by margins of *F*_1_-score=+0.045 and recall=+0.088 for instruction tuning and *F*_1_-score=+0.020 and recall=+0.024 for token classification.

**Table 2.** Performance of 17 encoder and decoder models in terms of weighted soft matching score on the J-CaseMap test set.^a^

Models^b^	*p*=1, mean (SD)			*p*=1.5, mean (SD)			*p*=2, mean (SD)			*p*=100, mean (SD)		
	*F*_1_-score	Recall	Precision	*F*_1_-score	Recall	Precision	*F*_1_-score	Recall	Precision	*F*_1_-score	Recall	Precision
Decoder models												
Instruction tuning												
GPT-Neox-3.6B	0.621 (0.023)	0.582 (0.044)	0.669 (0.006)	0.605 (0.022)	0.566 (0.042)	0.651 (0.005)	0.599 (0.022)	0.560 (0.042)	0.645 (0.005)	0.591 (0.022)	0.553 (0.041)	0.637 (0.005)
LLM-jp-13B v1.0	0.673 (0.011)	0.602 (0.022)	*0.763* *(0.007)* ^c^	0.660 (0.011)	0.588 (0.021)	*0.753* *(0.008)* ^c^	0.656 (0.011)	0.584 (0.021)	*0.750* *(0.008)* ^c^	0.651 (0.011)	0.577 (0.021)	*0.746* *(0.008)* ^c^
Swallow-7B	0.697 (0.009)	0.667 (0.037)	0.732 (0.027)	0.681 (0.007)	0.652 (0.037)	0.717 (0.032)	0.676 (0.006)	0.647 (0.037)	0.712 (0.034)	0.670 (0.005)	0.640 (0.037)	0.706 (0.036)
Swallow-13B	0.706 (0.010)	0.668 (0.024)	0.749 (0.016)	0.692 (0.011)	0.652 (0.024)	0.739 (0.019)	0.688 (0.011)	0.647 (0.023)	0.735 (0.020)	0.682 (0.011)	0.640 (0.023)	0.731 (0.021)
Swallow-70B	0.713 (0.016)	0.680 (0.035)	0.751 (0.020)	0.699 (0.015)	0.665 (0.034)	0.738 (0.023)	0.694 (0.015)	0.660 (0.034)	0.734 (0.024)	0.688 (0.015)	0.653 (0.034)	0.730 (0.025)
Swallow-MS-7B	0.693 (0.018)	0.648 (0.046)	0.747 (0.022)	0.679 (0.017)	0.633 (0.044)	0.735 (0.024)	0.675 (0.017)	0.628 (0.043)	0.732 (0.025)	0.669 (0.017)	0.621 (0.042)	0.727 (0.026)
Token classification												
GPTNeox-3.6B	0.725 (0.004)	0.731 (0.014)	0.719 (0.007)	0.698 (0.005)	0.683 (0.014)	0.714 (0.007)	0.689 (0.005)	0.668 (0.014)	0.713 (0.008)	0.678 (0.005)	0.649 (0.014)	0.710 (0.008)
LLM-jp-13B v1.0	0.732 (0.003)	0.742 (0.017)	0.722 (0.010)	0.707 (0.004)	0.696 (0.017)	0.718 (0.010)	0.698 (0.004)	0.681 (0.016)	0.716 (0.010)	0.688 (0.004)	0.663 (0.016)	0.714 (0.010)
Swallow-7B	0.732 (0.003)	0.733 (0.010)	0.731 (0.008)	0.706 (0.004)	0.686 (0.012)	0.727 (0.009)	0.697 (0.004)	0.671 (0.013)	0.725 (0.009)	0.686 (0.005)	0.653 (0.013)	0.723 (0.009)
Swallow-13B	0.735 (0.002)	0.739 (0.013)	0.730 (0.008)	0.710 (0.003)	0.696 (0.012)	0.725 (0.008)	0.702 (0.003)	0.683 (0.012)	0.724 (0.008)	0.692 (0.003)	0.666 (0.012)	0.721 (0.009)
Swallow-70B	0.739 (0.005)	0.744 (0.013)	0.734 (0.003)	0.714 (0.006)	0.698 (0.014)	0.730 (0.003)	0.706 (0.007)	0.684 (0.015)	0.729 (0.003)	0.695 (0.007)	0.666 (0.015)	0.727 (0.003)
Swallow-MS-7B	0.736 (0.002)	0.755 (0.010)	0.719 (0.008)	0.711 (0.002)	0.709 (0.009)	0.714 (0.008)	0.703 (0.002)	0.694 (0.009)	0.712 (0.008)	0.692 (0.002)	0.676 (0.009)	0.709 (0.008)
Encoder models												
Token classification												
DeBERTaV2-base	0.755 (0.006)	0.761 (0.015)	0.750 (0.006)	0.738 (0.007)	0.728 (0.016)	0.747 (0.006)	0.732 (0.007)	0.718 (0.016)	0.746 (0.006)	0.724 (0.008)	0.704 (0.017)	0.744 (0.006)
RoBERTa-base	0.748 (0.003)	0.748 (0.013)	0.749 (0.009)	0.729 (0.003)	0.713 (0.012)	0.746 (0.009)	0.722 (0.003)	0.701 (0.012)	0.745 (0.009)	0.714 (0.003)	0.687 (0.011)	0.743 (0.009)
JMedRoBERTa	0.756 (0.003)	*0.769* *(0.015)* ^c^	0.743 (0.009)	0.735 (0.003)	0.733 (0.015)	0.737 (0.010)	0.728 (0.003)	0.722 (0.016)	0.735 (0.010)	0.719 (0.004)	0.707 (0.016)	0.732 (0.011)
JMedDeBERTa(s)	*0.758* *(0.002)* ^d^ ^,^ ^c^	0.768 (0.011)	0.749 (0.009)	*0.740* *(0.002)* ^c^	*0.734* *(0.012)* ^c^	0.746 (0.010)	*0.734* *(0.002)* ^c^	0.724 (0.012)^c^	0.744 (0.010)	*0.726* *(0.002)* ^c^	*0.710* *(0.012)* ^c^	0.743 (0.010)
JMedDeBERTa(c)	0.757 (0.004)	0.764 (0.014)	0.751 (0.010)	0.739 (0.004)	0.730 (0.014)	0.748 (0.010)	0.732 (0.004)	0.719 (0.014)	0.747 (0.010)	0.724 (0.004)	0.705 (0.014)	0.746 (0.010)^c^

a *F*_1_-score, recall, and precision are shown for 4 conditions of the Hölder-type parameter *p* (1, 1.5, 2, 100), which controls the strength of the fragmentation penalty (*p*=1 corresponds to pure character-level evaluation; larger *p* penalizes split predictions of long target spans more heavily).

b Model performance is calculated using the weighted soft matching score, evaluating all extraction targets (clinical concepts) in the test set collectively.

c Highest score among the comparative models.

d Highest performance.

Next, we compared the performance of the decoder models. In the instruction tuning task, Swallow-70B achieved the highest mean *F*_1_-score 0.713 (SD 0.016) and mean recall 0.680 (SD 0.035), whereas GPT-NeoX-3.6B achieved the lowest mean *F*_1_-score 0.621 (SD 0.023) and mean recall 0.582 (SD 0.044). In the token classification task, Swallow-70B achieved the highest mean *F*_1_-score 0.739 (SD 0.005), and Swallow-MS-7B achieved the highest mean recall 0.755 (SD 0.010), whereas GPT-NeoX-3.6B achieved the lowest mean *F*_1_-score 0.725 (SD 0.004) and mean recall 0.731(SD 0.014). In the LLAMA2 decoder model, increasing parameters from 7 billion to 70 billion enhanced performance: *F*_1_-score +0.016 and recall +0.013 for the instruction tuning task, and *F*_1_-score +0.007 and recall +0.010 for the token classification task.

Finally, we compared the various encoder models. JMedDeBERTa(s) achieved the numerically highest mean *F*_1_-score (0.758, SD 0.002), and JMedRoBERTa achieved the highest mean recall (0.769, SD 0.015), whereas RoBERTa-base showed the lowest performance (mean *F*_1_-score 0.748, SD 0.003; and mean recall 0.748, SD 0.013). Notably, among encoder models, the DeBERTa family models (JMedDeBERTa(s), JMedDeBERTa(c), and DeBERTaV2-base) achieved the highest mean scores. However, differences within this top tier were marginal (Δ*F*_1_≤0.003 at *p*=1) and should be interpreted as effectively comparable, given the estimated annotation uncertainty (4% case-level corrections in our sampled review). DeBERTaV2-base achieved a higher *F*_1_-score (+0.007) than RoBERTa-base, and JMedDeBERTa(s) achieved a higher *F*_1_-score (+0.003) than JMedRoBERTa. JMedRoBERTa achieved a higher *F*_1_-score (+0.007) than RoBERTa-base.

### Performance on a Public Clinical NER Benchmark

Consistent with the E-CCR results on J-CaseMap, encoder models outperformed decoder models on the MRNER disease benchmark (Table S10 in Multimedia Appendix 1). The best-performing encoder model was DeBERTaV2-base, which achieved an mean *F*_1_-score of 0.794 (SD 0.018) at *p*=1, closely followed by JMedDeBERTa(c) (mean *F*_1_-score 0.788, SD 0.016) and JMedDeBERTa(s) (mean *F*_1_-score 0.788, SD 0.027). Among decoder models, LLM-jp-13B v1.0 in the token classification setting showed the highest performance (mean *F*_1_-score 0.694, SD 0.029), whereas instruction-tuned GPT-NeoX-3.6B performed worst (mean *F*_1_-score 0.373, SD 0.067). These results reinforce the main conclusion that, for Japanese clinical concept extraction, encoder-based token classification is consistently stronger than decoder-based approaches—whether instruction-tuned or token classification models—under the same span-level evaluation.

## Discussion

### Principal Findings

A key finding of this study is that, under the experimental conditions, encoder models consistently outperformed decoder models; no decoder model outperformed any encoder model.

On the J-CaseMap dataset, JMedDeBERTa(s)—an encoder model pretrained on domain-specific medical text—achieved the highest performance (*F*_1_-score=0.758, SD 0.002), whereas the instruction-tuned GPT-NeoX-3.6B produced the worst. While performance generally declined as the fragmentation penalty increased, no consistently severe degradation was observed. On the MRNER disease dataset, the general-domain DeBERTaV2-base yielded the highest mean *F*_1_-score. The differences among the medical-domain JMedDeBERTa(s) and JMedDeBERTa(c) variants were minimal, suggesting that the benefit of domain-specific pretraining was limited in this context. Overall, under the evaluated instruction tuning setup (a single prompt template requiring inline @@/## boundary tags), token classification encoders were more effective than instruction-tuned decoders in our experiments. However, because the generative setting additionally required faithful text reproduction plus precise character-level boundary-tag placement, the observed performance gap may partly reflect the difficulty of this particular output representation rather than model architecture alone. We did not evaluate alternative prompting strategies (eg, structured span lists or JSON outputs) or reasoning-augmented prompting (eg, chain-of-thought), which could affect generative performance. Importantly, encoder-based token classification is nonautoregressive and can assign labels to all tokens in parallel (ie, in a single forward pass), whereas decoder-based extraction via instruction tuning typically relies on autoregressive generation, whose runtime scales with the number of generated tokens. This architectural difference is expected to yield lower and more predictable inference latency and higher throughput for encoder-based token classification, which is particularly relevant for time-sensitive clinical workflows. Although we did not benchmark wall-clock inference latency in this study, we highlight this implication as a practical advantage of encoder-based approaches. Moreover, the smaller memory footprint of encoder models can make on-premise deployment more feasible when handling sensitive clinical text.

If, due to hallucination, the decoder extracts a string that differs from the original only in trivial ways—such as the insertion or deletion of a very small number of characters—then counting such negligible discrepancies as correct could, in principle, yield higher practical accuracy. However, as shown in Table S18 in Multimedia Appendix 1, after removing @@/## from both the gold-set inputs and the model outputs, the 5-fold mean exact match rate was 98.40% (SD 0.42%). Moreover, inspection of the mismatched cases indicated that most differences were minor: considering mismatches only, the mean SequenceMatcher similarity was 0.993 (SD 0.002), the mean Levenshtein distance was 7.54 (SD 1.99), and the mean length difference was −2.30 (SD 1.82). These findings suggest that even under a lenient assumption in which all such cases are treated as correct, the conclusion stated earlier would not change.

In recent years, the development of biomedical language models prioritized increasingly complex architectures and a rapid, often exponential growth in parameter count, in line with the consensus in LLM research [45,46]. However, some studies have reported that even a substantial increase in the parameter count of the decoder models does not necessarily improve model performance beyond that of encoder models [23,24], which is consistent with the findings of this study.

One possible explanation is that token classification constitutes a more constrained formulation of the prediction problem than free-form text generation via instruction tuning. Specifically, instead of having to localize the target span while generating fluent text, the model only needs to assign a binary label to each token. As the output space is thus more restricted, encoder models with far fewer parameters may plausibly compete with much larger decoder models. At the very least, a similar tendency was observed in both our main experiments and the additional experiments on the MRNER disease dataset. In our study, we demonstrated that even for tasks beyond conventional NER, such as E-CCR that targets longer phrases, encoder models with far fewer parameters are far more effective than decoder models. These findings offer valuable insight into model selection under practical constraints for general research institutions with limited computational resources, representing one of the substantial contributions of our work. Prior research has reported that decoder models require substantial computational resources and incur high costs, making them unsuitable when such resources are constrained [2].

Our goal was to compare off-the-shelf encoder versus decoder backbones under commonly used fine-tuning recipes, without altering the pretrained attention mechanism. An additional factor contributing to the encoder-decoder gap is attention directionality. Encoder models compute token representations bidirectionally, whereas decoder-only models under the default causal mask cannot incorporate future tokens when predicting token-level labels. This limitation may particularly affect boundary decisions in span labeling. Prior work indicates that partially or fully relaxing the causal mask (eg, layer-wise causal mask removal) can markedly improve sequence labeling with decoder-only LLM [42,43]. Under the standard fine-tuning recipes considered in this study, encoder backbones outperform decoder-only backbones on token classification; however, relaxing the causal mask (eg, via layer-wise mask removal) may improve decoder-only models and help narrow this gap, which we leave for future work.

Second, a comparison of the evaluated decoder models showed that instruction-tuned variants consistently underperformed their token classification counterparts. This contrasts with prior work [28], which suggested that decoder models can match or even surpass encoder models when supported by carefully designed prompts. Our results indicate that, even within decoder architectures, token classification is more effective than instruction tuning for extraction tasks. Although we did not exhaustively explore all prompt designs, the E-CCR task requires extracting entire phrases corresponding to single clinical concepts. This is substantially more complex than conventional NER, making effective prompt design for instruction tuning considerably more challenging. Overall, these findings suggest that token classification provides a more robust approach for specialized extraction scenarios across both encoder and decoder models.

Among the various token classification–based encoder models investigated in this study, JMedDeBERTa(s) achieved the highest mean performance, whereas RoBERTa-base performed the worst. However, the differences among the top encoder models were very small and should be interpreted as comparable within the uncertainty implied by our annotation review. This is consistent with previous research [34] and indicates that the DeBERTa architecture is more effective than the RoBERTa architecture. In particular, DeBERTa uses the disentangled attention mechanism that allows for a more precise interpretation of the importance of word positions and the contextual relationships among words, as well as a masked decoder that predicts not only the masked tokens but also their positions [47,48]. These mechanisms likely enhance the understanding of linguistic structure and context, resulting in high performance even on E-CCR tasks.

With respect to the effect of domain-specific text versus general text, no significant difference was detected; however, the highest accuracy was achieved by the model pretrained on domain-specific documents, followed by the model that underwent additional pretraining on a generally pretrained model, and finally by the model pretrained solely on general text. For example, among the DeBERTa models, JMedDeBERTa(s)—pretrained on medical documents—outperformed both DeBERTaV2-base (pretrained on general Japanese texts) and JMedDeBERTa(c), which was additionally pretrained on medical records. Although JMedDeBERTa(c) performed better than DeBERTaV2-base, it did not reach the performance level of JMedDeBERTa(s). Similarly, among the RoBERTa models, MedRoBERTa, which was pretrained on medical documents, outperformed RoBERTa-base, which was pretrained on general Japanese texts. However, the differences were minimal, indicating that the distinctions among these pretraining approaches are negligible.

### Comparison to Prior Work

This observation aligns with previous studies. Kim et al [49] reported that a model pretrained on domain-specific Korean medical documents outperformed a general Korean language model. Conversely, Subies et al [33] found that a model pretrained on domain-specific Spanish medical records did not surpass the performance of a model pretrained on general Spanish texts or that of a multilingual model. As with our findings, the differences in model comparison results in these studies were minimal.

Similar to the minimal performance differences reported by Kim et al [49] and Subies et al [33], the slight differences in performance observed in our study may be attributed to the quantity and content of the texts used for both pretraining and additional pretraining. Specifically, only 0.56 GB of medical text was used to train the domain-specific models JMedDeBERTa(c) and JMedDeBERTa(s), compared to the 362 GB of text used to train the general-corpus DeBERTaV2-base model.

Although the pretraining corpus is small compared with typical general-language pretraining datasets, the MLM loss curves for JMedDeBERTa(s) and JMedDeBERTa(c) (Figures S1 and S2 in Multimedia Appendix 1) indicate stable optimization and convergence under our setting. We note, however, that convergence of the MLM loss does not by itself guarantee broad linguistic coverage or robustness across institutions and document types. A possible reason why effective learning may be achievable with a relatively small corpus is the qualitative nature of discharge summaries: compared with fragmented note types (eg, brief progress notes), discharge summaries are often more narrative and syntactically coherent, potentially enabling MLM pretraining to capture clinically salient vocabulary and patterns efficiently. Our findings indicate that, at least for the E-CCR task examined in this study, even a model pretrained on a very small domain-specific corpus can achieve performance comparable to, or slightly exceeding, that of a model trained on a large general corpus. This highlights the high data efficiency of domain-specific pretraining in this setting: even a small amount of domain-specific text may be sufficient to obtain performance on par with a model trained on a much larger general corpus. In contrast, our results also indicate that, even when only a limited amount of domain-specific text is available, a model trained solely on general text may still achieve satisfactory performance on the E-CCR task. This is presumably because, in the E-CCR task, the syntactic characteristics of the text play a crucial role even when specialized terminology has not been thoroughly acquired. For example, even if the model has not explicitly learned each specialized term, when processing a sentence such as “SAA was abnormally elevated to 1940 mg/mL, and FMF was suspected,” it may still be able to extract spans such as “SAA was abnormally elevated to 1940 mg/mL” and “FMF” by using syntactic information as clues. Therefore, training strategies should be flexibly adjusted according to the amount and type of data available.

### External Benchmark and Implications for Domain-Specific Versus General Pretraining

Additional experiments using the MRNER disease dataset further support these conclusions. Even in this conventional clinical NER task, encoder models consistently outperformed decoder models; however, the general-domain DeBERTaV2-base slightly outperformed the domain-specific JMedDeBERTa(s) (eg, *F*_1_=0.794, SD 0.018 vs 0.788, SD 0.027 at *p*=1). However, this difference was marginal and likely reflects multiple factors.

We interpret this result not as a lack of generalization ability in JMedDeBERTa(s) but primarily as a reflection of task mismatch and distribution shift. First, MRNER disease is a conventional clinical NER task targeting short disease name entities, whereas E-CCR, our primary evaluation target, focuses on longer, more complex, and clinically meaningful spans that often exceed typical NER boundaries. Therefore, the relative advantage of domain-specific versus general-domain pretraining can vary depending on specific task requirements. Additionally, distribution shifts arising from differences in document types, medical institutions, and annotation criteria may have also influenced these results.

The substantial disparity in the size and composition of pretraining data (362 GB vs 0.56 GB) further complicates this comparison. Although JMedDeBERTa(s) achieved the highest average performance on the E-CCR task, the differences among the top-tier encoder models were generally comparable within the margin of uncertainty suggested by our annotation review. Taken together, these findings suggest that while pretraining on a massive general-domain corpus provides a solid linguistic foundation, pretraining on a much smaller domain-specific corpus can achieve competitive performance—comparable to or slightly exceeding general-domain models—depending on the task constraints and linguistic characteristics of the target documents.

However, as the external benchmark consists of only 100 documents, these slight performance differences are not necessarily robust and should be interpreted with caution. Moreover, previous research has reported substantially low zero-shot and few-shot performance of pretrained LLMs on MRNER disease (entity-level *F*_1_ ≤0.3). In contrast, the significantly higher scores achieved in this study highlight the critical importance of supervised fine-tuning in Japanese clinical NER, demonstrating that merely prompting non–fine-tuned LLMs is insufficient for achieving adequate performance in this context.

### Differences in Characteristics Between Instruction Tuning and Token Classification

The differences in the properties of instruction tuning and token classification were analyzed from the perspective of the length of the extracted segments. Table S1 in Multimedia Appendix 1 presents evaluation results using the marker matching score, which does not account for the length of the extraction targets. In contrast to Table 2, where longer extracted segments yield higher scores, Table S1 in Multimedia Appendix 1 simply counts each extraction as one regardless of its length. Consequently, frequently occurring short terms (eg, “lung cancer”) that are repeatedly extracted may appear to achieve a high score.

Comparing Table 2 and Table S1 in Multimedia Appendix 1 reveals that, for instruction tuning, all models except GPT-NeoX-3.6B attained higher *F*_1_-scores in Table S1 in Multimedia Appendix 1.

In addition, we stratified the gold entities into 5 character-length bins (1‐2, 3‐6, 7‐10, 11‐20, and ≥21) and computed precision, recall, and *F*_1_-score within each bin (Tables S19-S21 in Multimedia Appendix 1). Under the weighted soft matching score with *p*=1 (equivalent to character-level evaluation), the decoder model (Swallow-70B) achieved the highest *F*_1_-score for the shortest spans (1‐2 characters; *F*_1_-score=0.814, SD 0.017), but its performance dropped rapidly as the span length increased (≥21 characters; *F*_1_-score=0.677, SD 0.015). In contrast, the encoder model (JMedDeBERTa(s)) showed relatively low *F*_1_-score for the extremely short spans (1‐2 characters), which account for only 9% of the dataset, while consistently outperforming the decoder model for spans of 3 characters or longer and maintaining robust performance even for long spans (≥10 characters). A similar trend was also observed in the stratified analysis based on the marker matching score, which does not weight span length.

This suggests that instruction tuning is effective for extracting short terms but less so for long expressions. When using instruction tuning, both sides of the extraction segments must be enclosed with markers. For E-CCR, which includes long phrases, the attention mechanism must retain these extended, marker-enclosed segments. This requirement may account for the discrepancy with a previous study [28] that reported superior performance for instruction tuning compared with encoder models when extracting terms or short phrases (eg, disease names or gene identifiers). The fact that encoder models outperformed decoder models for spans of 3 characters or longer—which constitute the majority (91%) of entities in E-CCR—is also clinically meaningful.

A comparison of decoder models using token classification reveals higher *F*_1_-scores in Table 2 than in Table S1 in Multimedia Appendix 1. This suggests that token classification remains effective for extracting long phrases. When using token classification, the model assigns a 0 or 1 to each token, and this token-level information can be used in subsequent outputs, potentially making it easier to handle long markers than instruction tuning.

When long expressions are extracted, unintended portions of the text may also be included, potentially reducing precision. However, in applications such as automated knowledge graph construction, such noise is not critical, as it can be filtered out during subsequent processing (eg, normalization). The key point is that the relatively long expressions that need to be extracted are reliably captured. Token classification achieved a higher recall than instruction tuning and is therefore well suited to applications where E-CCR is followed by a series of postprocessing steps, such as normalization of the extracted segments and causal inference among terms, as in automatic knowledge graph construction.

Seventeen models were compared using the weighted soft matching score, which accounts for penalties when the extracted segments are fragmented. In particular, when *p*=1, the evaluation is equivalent to that based on character-level segmentation. Across all *p *values, token classification consistently outperformed instruction tuning, and encoder models outperformed decoder models. The scores did not decrease substantially at higher *p*, suggesting that cases in which the marker positions become fragmented during extraction are rare.

**Figure 6.**
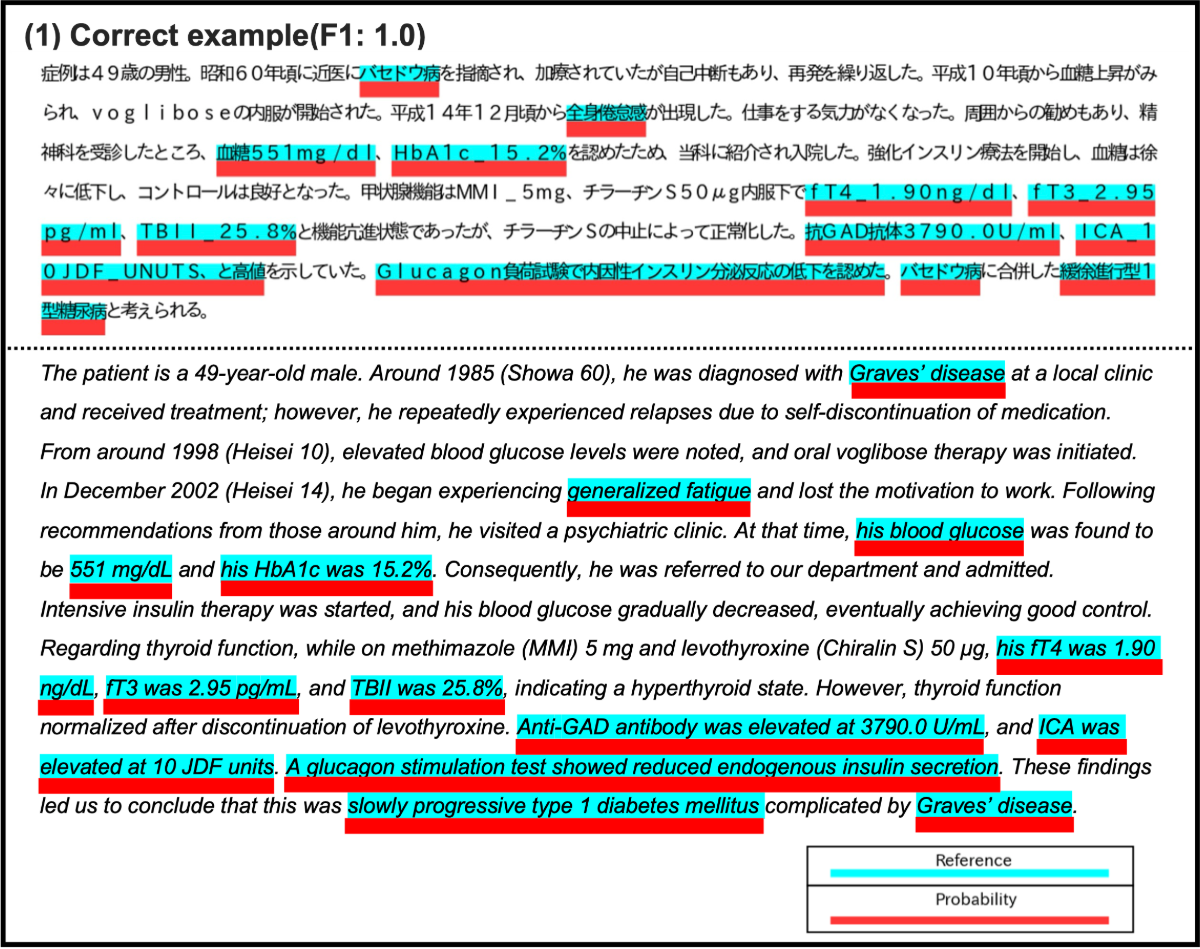
Example of perfect extraction by JMedDeBERTa(s) in the test set. The upper row shows the original Japanese case report text, and the lower row shows the corresponding English translation. Blue highlights indicate gold-standard clinically essential expressions, and red highlights indicate spans predicted by JMedDeBERTa(s) fine-tuned with token classification. In this example, the predicted spans exactly match the gold standard, resulting in a weighted soft matching F1 score of 1.0.

Figure 6 shows an example of the output results (for the test set) of JMedDeBERTa(s), which exhibited the best performance. The output results include (1) cases in which the extraction of critical clinical concepts was performed correctly and (2) cases in which the extraction of critical clinical concepts failed. In particular, in (2), as shown in Figure 6, cases exist where the model does not extract the part that should be extracted; however, cases also exist where the output results are not necessarily wrong, as shown in Figures S3-S5 in Multimedia Appendix 1, and cases exist where the *F*_1_-score decreases because of labeling-related problems. Therefore, the actual model performance may be underestimated and warrants further investigation in future research.

To gauge the potential impact of such labeling issues on performance evaluation, we randomly sampled 200 cases from the test set and reassessed the correspondence between the gold-standard labels and the original case report texts. As a result, we found that in 8 of the 200 cases (4%), at least one gold-standard label required correction; in most of these cases, the problem was attributable not to completely inappropriate concepts, but rather to omissions of important expressions that should have been included or to deviations in span boundaries. Thus, although the overall quality of the gold standard appears to be generally high, the actual model performance may be slightly underestimated, and a more systematic investigation of this issue will be needed in future work. These findings suggest that the measured *F*_1_-score may be partially bounded by residual label noise and annotation subjectivity, potentially constituting a performance ceiling in agreement with the reference standard.

### Novelty of This Study

This study is the first to compare instruction tuning and token classification and decoder and encoder models, in the context of the comprehensive E-CCR task.

In clinical concept extraction, capturing both long and conventional short expressions is essential. This study extended conventional NER, which typically targets words or short phrases, by proposing an E-CCR task that includes long expressions as extraction targets together with a weighted soft matching score. This extension represents a significant contribution of this work. To the best of our knowledge, few studies have focused on extracting long expressions to the extent achieved in this study. The study by Chen et al [15] addressed long expression extraction by summarizing discharge summaries through segment identification from the texts using AlphaBERT. Although Chen et al focused on extracting expressions representing single clinical concepts with AlphaBERT, their evaluation was limited to character-level analysis and did not incorporate metrics penalizing fragmented extraction. In contrast, the E-CCR framework explicitly includes long expressions as extraction targets. Chen et al.’s study was limited to comparative evaluations among AlphaBERT, BERT, BioBERT, and LSTM models, without incorporating analyses using the rapidly advancing decoder models. The inclusion of comparative evaluations using various decoder models alongside encoder models constitutes a significant advancement.

### Future Directions

E-CCR can be applied not only to knowledge extraction for diagnostic support but also to other extraction tasks involving relatively long expressions. For example, consider a case in which information on daily living functions is extracted from a text and coded using the International Classification of Functioning, Disability and Health. When making a judgment of “嚥下機能低下 (中等度)”<b5105.2> (“swallowing function decline (moderate)”<b5105.2>) in response to the expression “嚥下造影検査:反射惹起遅延(+)。液体で少量の誤嚥あり, ペースト食で少量の残留あり。咳反射あるが微弱” (“swallowing contrast study: reflex-induced delay (+). Small amount of aspiration with liquid, small amount of residual with paste food. Cough reflex present but weak”), the part corresponding to b5105.2 must be extracted, which is not a single word but a single description that spans multiple sentences. Additionally, numerous other applications can be envisioned, such as International Statistical Classification of Diseases and Related Health Problems coding of patient condition descriptions in clinical text, thereby providing valuable insights for extraction tasks across a wide range of medical texts.

### Limitations

First, all experiments were conducted on Japanese clinical case reports. As Japanese differs substantially from English and other languages that dominate clinical natural language processing research—most notably in the lack of whitespace and the use of mixed scripts—our findings may not directly generalize to other languages or document types.

Second, for instruction-tuned decoder-based models, we evaluated only a single prompt template and a single instruction or output format, following prior work [23,28], in which extraction spans are generated as inline boundary tags using the @@ and ## markers. We did not perform a systematic prompt engineering study (eg, ablations over instruction wording, formatting, or response schemas). As LLM extraction performance is sensitive to both prompt design and output representation, alternative prompting strategies and constrained output formats—such as returning a structured list of extracted spans, structured JSON with character offsets, or other boundary representations—may lead to different results. We also did not explore reasoning-augmented prompting (eg, rationale or chain-of-thought prompting), including variants where reasoning is elicited but the final answer is constrained to a structured schema, which could affect generative extraction behavior. Thus, the observed performance gap between encoder and decoder models may partly reflect the difficulty of generating precise character-level boundary tags under this specific template or format. Future work should systematically compare multiple prompting or output strategies to disentangle the effects of model architecture from output format constraints.

Third, to maintain consistent label-token alignment across models, we disabled byte fallback. Consequently, out-of-vocabulary characters were normalized to <unk>, potentially discarding surface cues for rare kanji and specialized medical symbols. This may have disadvantaged models with smaller vocabularies, including GPT-NeoX-3.6B, DeBERTaV2-base, RoBERTa-base, and the JMedRoBERTa/JMedDeBERTa variants. Notably, however, all models used for token classification—both decoder only and encoder based—were evaluated under this same unfavorable setting (ie, with byte fallback disabled), yet the token classification still outperformed instruction tuning overall.

Fourth, owing to computational constraints, we applied parameter-efficient fine-tuning with LoRA to decoder models, whereas encoder models were fully fine-tuned. Although LoRA has been reported to achieve performance comparable to full fine-tuning [41], its effectiveness can vary depending on the task and hyperparameter settings; therefore, caution is warranted when interpreting these comparative results.

Fifth, for decoder-only models in token classification, we retained the default causal attention mask; therefore, token-level predictions could not leverage right-side context. As bidirectional information is often helpful for sequence labeling, this design may disadvantage causal-masked decoders relative to encoders. Approaches that enable bidirectional information in decoder-only LLM (eg, layer-wise causal mask removal) have been reported to improve sequence labeling performance [42,43]. Therefore, our conclusion that encoder-based token classification outperformed decoder-based approaches should be interpreted as holding for decoder-only models used with the default causal mask under our fine-tuning constraints and may not generalize to decoder variants explicitly adapted to incorporate right-context information.

Sixth, comparisons between general-domain and medical-domain pretraining are confounded by substantial differences in corpus size (362 GB vs 0.56 GB) and data composition. The strong performance of general-domain models—despite limited exposure to specialized medical terminology—may reflect not only syntactic knowledge but also other advantages conferred by massive training data. Rigorous ablation studies that control for architecture, corpus size, and training conditions are therefore needed. Further research is also required to determine whether the observed encoder-decoder performance gap, the relative benefits of domain-specific versus general-domain pretraining, and the effects of prompting strategies generalize to other languages, clinical settings, and medical document types.

Seventh, the weighted soft matching score is an automated evaluation metric whose clinical validity has not yet been validated, including its alignment with clinician judgments, and its usefulness for downstream tasks. Moreover, as the score is computed based on the span boundaries of the reference annotations, it may penalize predictions even when boundary differences are clinically acceptable. Future work should quantify correlations with clinician ratings and downstream task performance to establish the metric’s clinical validity.

Finally, our reference annotations represent an adjudicated consensus rather than an objective ground truth. As preadjudication annotations were not retained, we cannot report IAA to quantify the inherent subjectivity of the task. In a post hoc audit, 8 (4%) of 200 randomly sampled test cases contained at least one span requiring correction, mostly due to omissions or boundary deviations, indicating residual label noise and subjective boundary decisions. Moreover, as illustrated by examples where model predictions are clinically plausible despite differing from the reference, span-level *F*_1_-score should be interpreted as agreement with this consensus reference, and may be bounded by a ceiling on reference-based performance rather than model capability. This limitation primarily affects the absolute magnitude of the scores; however, as all models were evaluated against the same reference standard under the same metric, relative comparisons across models remain informative. Future work will retain independent preadjudication labels to compute IAA and will complement reference-based metrics with clinician-centered acceptability assessments and/or more tolerant scoring schemes.

### Conclusions

This study is the first to investigate effective model selection for the E-CCR task—an extraction task targeting terms and extended expressions (eg, diseases, conditions, and clinical findings considered critical for differential diagnosis) that are indispensable for the automatic construction of causal relationship knowledge from case reports—by comparing encoder versus decoder models, as well as general-purpose versus domain-specific models. Additionally, we introduced a novel evaluation metric tailored to the E-CCR task, the weighted soft matching score. Furthermore, we analyzed model effectiveness in relation to the length of the extraction targets.

As a result, encoder-based token classification achieved the best performance in our evaluation. Under our evaluation setting—where decoder models were constrained to output entity spans using inline boundary tags (eg, @@ and ##)—token classification generally outperformed instruction tuning. Moreover, among the 17 models compared, we observed no case where a decoder model outperformed its corresponding encoder model. These findings suggest that, for the E-CCR task, token classification–based extraction with encoder models can achieve high accuracy with relatively fewer parameters, making it advantageous in resource-constrained environments. This efficiency is also beneficial for real-world deployment in clinical practice, where inference latency and throughput are critical. That said, although encoder token classification outperformed decoder approaches under our standard fine-tuning recipe, decoder performance may improve with alternative prompting strategies, the use of reasoning-oriented models (ie, increased inference-time computation), or architectural or training modifications such as layer-wise relaxation of the causal mask for instruction tokens.

In contrast, while JMedDeBERTa(s), pretrained on medical text, obtained the highest average score on the E-CCR task, DeBERTaV2-base achieved the highest average score on the MRNER disease benchmark. These results indicate that there is no single universally optimal model; the relative advantage of domain-specific versus general-domain pretraining likely depends on the characteristics of the target task and the dataset.

Our results examining the effect of domain-specific texts relative to general texts align with previous findings [33], indicating only marginal performance differences between models trained with and without domain-specific texts.

Furthermore, our findings indicate that models trained on relatively small amounts of domain-specific text can match or exceed the performance of models trained on large general corpora. Conversely, in situations where domain-specific text is scarce, models trained solely on general corpora may still deliver high performance. Therefore, model selection should be guided by the quantity and characteristics of the available training texts.

An analysis of the differences between instruction tuning and token classification with respect to the length of the extracted segments revealed that instruction tuning is well suited for extracting short terms but is less effective for extracting long expressions. In contrast, token classification proved effective for extracting extended phrases. Furthermore, evaluation using the weighted soft matching score showed that increasing the fragmentation penalty did not substantially degrade model performance. This suggests that marker positions split during extraction are infrequent, even when extracting long expressions.

A detailed analysis of the outputs from JMedDeBERTa(s) revealed inaccuracies in some manually assigned correct labels. This observation implies that actual model accuracy may surpass the reported figures.

Extracting clinical concepts necessitates targeting both conventional short expressions and extended phrases. To our knowledge, few prior studies have comprehensively addressed extended expression extraction in clinical texts. In this study, we performed model comparisons on a more comprehensive E-CCR task using the weighted matching score, obtaining insights into model selection, effectiveness with respect to expression length, and the impact of domain-specific texts. These findings constitute a substantial contribution to the advancement of clinical information extraction methodologies.

## Supplementary material

10.2196/78681 Multimedia Appendix 1 Extended experimental details and evaluation results that support the main findings. Contents include (1) overall and case-level performance of the 17 encoder or decoder models on the J-CaseMap and MRNER disease datasets under 4 scoring metrics (weighted soft matching, marker matching, character segment, and token segment scores), (2) pretraining and fine-tuning hyperparameters for JMedDeBERTa(s) or JMedDeBERTa(c) and all comparison models, (3) training and validation loss curves, (4) input-output agreement analysis for the instruction-tuned decoder, (5) gold entity length distribution and length-stratified performance, and (6) representative examples illustrating discrepancies between model predictions and reference annotations.

## Abbreviations

BERT: Bidirectional Encoder Representations from Transformers
E-CCR: extended clinical concept recognition
IAA: interannotator agreement
LLM: large language model
LoRA: low-rank adaptation
LSTM: long short-term memory
MLM: masked language modeling
MRNER disease: Medical Report Named Entity Recognition for positive disease
NER: Named Entity Recognition
