# Supplementary material for: Evaluating Encoder and Decoder Models for Extended Clinical Concept Recognition in Japanese Clinical Texts: Comparative Study With Weighted Soft Matching
Source: J Med Internet Res. 2026 May 14;28:e78681. doi: 10.2196/78681 (PMC13175525; doi:10.2196/78681)

## Appendix

Table S1. Marker matching score performance of 17 encoder/decoder models on the J-CaseMap test set. Performance is calculated using the marker matching score, evaluating all extraction targets collectively. In this metric, each annotated clinical concept (marker span) is treated as 1 unit regardless of its length. F1, Recall, and Precision are shown for 4 conditions of the fragmentation penalty parameter p (1, 1.5, 2, 100) for each model. Values are presented as mean (SD).

| J-CaseMap  All Extraction Targets | p=1 | | | p=1.5 | | | p=2 | | | p=100 | | |
| --- | --- | --- | --- | --- | --- | --- | --- | --- | --- | --- | --- | --- |
|  | F1 | Recall | Precision | F1 | Recall | Precision | F1 | Recall | Precision | F1 | Recall | Precision |
| Decoder Models |  |  |  |  |  |  |  |  |  |  |  |  |
| Instruction Tuning |  |  |  |  |  |  |  |  |  |  |  |  |
| GPTNeox-3.6B | 0.574  (0.020) | 0.643  (0.042) | 0.519  (0.006) | 0.571  (0.019) | 0.639  (0.042) | 0.516  (0.005) | 0.569  (0.019) | 0.638  (0.042) | 0.515  (0.005) | 0.567  (0.019) | 0.636  (0.041) | 0.513  (0.005) |
| LLM-jp-13B v1.0 | 0.708  (0.008) | 0.657  (0.017) | **0.768**  **(0.007)** | 0.705  (0.008) | 0.653  (0.017) | **0.766**  **(0.007)** | 0.704  (0.008) | 0.652  (0.017) | **0.766**  **(0.007)** | 0.703  (0.008) | 0.650  (0.017) | **0.765**  **(0.007)** |
| Swallow-7B | 0.731  (0.013) | 0.723  (0.031) | 0.740  (0.022) | 0.727  (0.013) | 0.719  (0.031) | 0.738  (0.022) | 0.726  (0.013) | 0.717  (0.031) | 0.737  (0.022) | 0.725  (0.013) | 0.715  (0.031) | 0.736  (0.022) |
| Swallow-13B | 0.740  (0.010) | 0.724  (0.028) | 0.757  (0.013) | 0.737  (0.010) | 0.720  (0.028) | 0.756  (0.013) | 0.736  (0.010) | 0.719  (0.028) | 0.755  (0.013) | 0.734  (0.010) | 0.716  (0.028) | 0.754  (0.013) |
| Swallow-70B | **0.747**  **(0.017)** | 0.731  (0.038) | 0.765  (0.007) | **0.744**  **(0.017)** | 0.727  (0.038) | 0.763  (0.007) | **0.743**  **(0.017)** | 0.726  (0.038) | 0.762  (0.007) | **0.741**  **(0.017)** | 0.723  (0.038) | 0.762  (0.007) |
| Swallow-MS-7B | 0.726  (0.018) | 0.696  (0.048) | 0.761  (0.017) | 0.723  (0.018) | 0.692  (0.047) | 0.759  (0.017) | 0.722  (0.017) | 0.691  (0.047) | 0.759  (0.017) | 0.720  (0.017) | 0.689  (0.047) | 0.758  (0.017) |
| Token Classification |  |  |  |  |  |  |  |  |  |  |  |  |
| GPTNeox-3.6B | 0.675  (0.004) | 0.798  (0.012) | 0.584  (0.006) | 0.668  (0.004) | 0.781  (0.012) | 0.584  (0.006) | 0.665  (0.004) | 0.775  (0.012) | 0.583  (0.006) | 0.662  (0.004) | 0.767  (0.012) | 0.583  (0.006) |
| LLM-jp-13B v1.0 | 0.678  (0.003) | 0.813  (0.017) | 0.582  (0.010) | 0.672  (0.003) | 0.797  (0.018) | 0.581  (0.010) | 0.670  (0.002) | 0.791  (0.018) | 0.581  (0.010) | 0.667  (0.002) | 0.784  (0.018) | 0.581  (0.010) |
| Swallow-7B | 0.682  (0.004) | 0.801  (0.008) | 0.593  (0.010) | 0.675  (0.004) | 0.784  (0.009) | 0.593  (0.010) | 0.672  (0.004) | 0.778  (0.009) | 0.592  (0.010) | 0.669  (0.004) | 0.770  (0.009) | 0.592  (0.010) |
| Swallow-13B | 0.680  (0.003) | 0.803  (0.014) | 0.590  (0.009) | 0.674  (0.003) | 0.788  (0.014) | 0.589  (0.009) | 0.672  (0.003) | 0.783  (0.014) | 0.589  (0.010) | 0.669  (0.003) | 0.776  (0.014) | 0.588  (0.010) |
| Swallow-70B | 0.688  (0.003) | 0.812  (0.013) | 0.597  (0.003) | 0.682  (0.003) | 0.797  (0.014) | 0.597  (0.003) | 0.680  (0.004) | 0.791  (0.014) | 0.596  (0.003) | 0.677  (0.004) | 0.784  (0.014) | 0.596  (0.003) |
| Swallow-MS-7B | 0.677  (0.002) | 0.819  (0.013) | 0.577  (0.007) | 0.671  (0.002) | 0.803  (0.013) | 0.576  (0.007) | 0.669  (0.002) | 0.798  (0.013) | 0.576  (0.007) | 0.666  (0.002) | 0.791  (0.013) | 0.575  (0.007) |
| Encoder Models |  |  |  |  |  |  |  |  |  |  |  |  |
| Token Classification |  |  |  |  |  |  |  |  |  |  |  |  |
| DeBERTaV2-base | 0.746  (0.009) | 0.834  (0.013) | 0.675  (0.018) | 0.742  (0.009) | 0.824  (0.013) | 0.674  (0.018) | 0.740  (0.009) | 0.821  (0.013) | 0.674  (0.018) | 0.738  (0.010) | 0.817  (0.013) | 0.673  (0.018) |
| RoBERTa-base | 0.740  (0.004) | 0.819  (0.018) | 0.675  (0.013) | 0.736  (0.004) | 0.810  (0.018) | 0.675  (0.013) | 0.734  (0.004) | 0.806  (0.018) | 0.674  (0.013) | 0.732  (0.004) | 0.801  (0.018) | 0.674  (0.013) |
| JMedRoBERTa | 0.746  (0.001) | 0.838  (0.014) | 0.673  (0.011) | 0.741  (0.001) | 0.828  (0.014) | 0.672  (0.011) | 0.740  (0.001) | 0.824  (0.015) | 0.671  (0.011) | 0.737  (0.001) | 0.819  (0.015) | 0.670  (0.011) |
| JMedDeBERTa(s) | 0.742  (0.003) | **0.842**  **(0.010)** | 0.664  (0.010) | 0.738  (0.003) | **0.832**  **(0.010)** | 0.663  (0.010) | 0.736  (0.003) | **0.829**  **(0.010)** | 0.663  (0.010) | 0.734  (0.003) | **0.824**  **(0.010)** | 0.662  (0.010) |
| JMedDeBERTa(c) | 0.735  (0.005) | 0.836  (0.016) | 0.656  (0.013) | 0.731  (0.005) | 0.826  (0.016) | 0.656  (0.013) | 0.729  (0.005) | 0.822  (0.016) | 0.656  (0.013) | 0.727  (0.005) | 0.817  (0.017) | 0.655  (0.013) |

Table S2. Character-level performance (character segment score) of 17 encoder/decoder models on the J-CaseMap test set. Model performance is calculated on a character-by-character basis. F1, Recall, and Precision are shown. Values are presented as mean (SD).

| J-CaseMap  All Extraction Targets | F1 | Recall | Precision |
| --- | --- | --- | --- |
|  |  |  |  |
| Decoder Models |  |  |  |
| Instruction Tuning |  |  |  |
| GPTNeox-3.6B | 0.621  (0.023) | 0.582  (0.044) | 0.669  (0.006) |
| LLM-jp-13B v1.0 | 0.673  (0.011) | 0.602  (0.022) | **0.763**  **(0.007)** |
| Swallow-7B | 0.697  (0.009) | 0.667  (0.037) | 0.732  (0.027) |
| Swallow-13B | 0.706  (0.010) | 0.668  (0.024) | 0.749  (0.016) |
| Swallow-70B | 0.713  (0.016) | 0.680  (0.035) | 0.751  (0.020) |
| Swallow-MS-7B | 0.693  (0.018) | 0.648  (0.046) | 0.747  (0.022) |
| Token Classification |  |  |  |
| GPTNeox-3.6B | 0.725  (0.004) | 0.731  (0.014) | 0.719  (0.007) |
| LLM-jp-13B v1.0 | 0.732  (0.003) | 0.742  (0.017) | 0.722  (0.010) |
| Swallow-7B | 0.732  (0.003) | 0.733  (0.010) | 0.731  (0.008) |
| Swallow-13B | 0.735  (0.002) | 0.739  (0.013) | 0.730  (0.008) |
| Swallow-70B | 0.739  (0.005) | 0.744  (0.013) | 0.734  (0.003) |
| Swallow-MS-7B | 0.736  (0.002) | 0.755  (0.010) | 0.719  (0.008) |
| Encoder Models |  |  |  |
| Token Classification |  |  |  |
| DeBERTaV2-base | 0.755  (0.006) | 0.761  (0.015) | 0.750  (0.006) |
| RoBERTa-base | 0.748  (0.003) | 0.748  (0.013) | 0.749  (0.009) |
| JMedRoBERTa | 0.756  (0.003) | **0.769**  **(0.015)** | 0.743  (0.009) |
| JMedDeBERTa(s) | **0.758**  **(0.002)** | 0.768  (0.011) | 0.749  (0.009) |
| JMedDeBERTa(c) | 0.757  (0.004) | 0.764  (0.014) | 0.751  (0.010) |

Table S3. Token-level performance (token segment score) of 17 encoder/decoder models on the J-CaseMap test set. Model performance is calculated on a token-by-token basis. F1, Recall, and Precision are shown. Values are presented as mean (SD).

| J-CaseMap  All Extraction Targets | F1 | Recall | Precision |
| --- | --- | --- | --- |
|  |  |  |  |
| Decoder Models |  |  |  |
| Instruction Tuning |  |  |  |
| GPTNeox-3.6B | 0.630  (0.023) | 0.598  (0.045) | 0.669  (0.005) |
| LLM-jp-13B v1.0 | 0.676  (0.012) | 0.606  (0.022) | 0.763  (0.007) |
| Swallow-7B | 0.702  (0.009) | 0.674  (0.036) | 0.734  (0.026) |
| Swallow-13B | 0.711  (0.010) | 0.675  (0.024) | 0.752  (0.015) |
| Swallow-70B | 0.718  (0.016) | 0.687  (0.036) | 0.754  (0.019) |
| Swallow-MS-7B | 0.698  (0.019) | 0.654  (0.047) | 0.751  (0.021) |
| Token Classification |  |  |  |
| GPTNeox-3.6B | 0.734  (0.005) | 0.724  (0.013) | 0.744  (0.006) |
| LLM-jp-13B v1.0 | 0.735  (0.005) | 0.724  (0.019) | 0.747  (0.011) |
| Swallow-7B | 0.736  (0.003) | 0.718  (0.010) | 0.756  (0.007) |
| Swallow-13B | 0.738  (0.004) | 0.724  (0.014) | 0.752  (0.009) |
| Swallow-70B | 0.744  (0.005) | 0.731  (0.014) | 0.758  (0.004) |
| Swallow-MS-7B | 0.739  (0.002) | 0.740  (0.011) | 0.738  (0.008) |
| Encoder Models |  |  |  |
| Token Classification |  |  |  |
| DeBERTaV2-base | 0.769  (0.006) | 0.773  (0.015) | 0.765  (0.005) |
| RoBERTa-base | 0.761  (0.003) | 0.757  (0.014) | 0.765  (0.009) |
| JMedRoBERTa | 0.754  (0.004) | 0.757  (0.015) | 0.752  (0.009) |
| JMedDeBERTa(s) | 0.755  (0.002) | 0.753  (0.011) | 0.758  (0.009) |
| JMedDeBERTa(c) | **0.771**  **(0.004)** | **0.774**  **(0.016)** | **0.768**  **(0.009)** |

Table S4. Case-level Weighted Soft Matching Score for 17 encoder/decoder models on the J-CaseMap test set. F1, Recall, and Precision scores were calculated for all extraction targets (clinical concepts) for each case report and averaged. Results are shown for 4 conditions of the fragmentation penalty parameter p (1, 1.5, 2, 100). Values are presented as mean (SD).

| J-CaseMap  Each Extraction Targets | p=1 | | | p=1.5 | | | p=2 | | | p=100 | | |
| --- | --- | --- | --- | --- | --- | --- | --- | --- | --- | --- | --- | --- |
|  | F1 | Recall | Precision | F1 | Recall | Precision | F1 | Recall | Precision | F1 | Recall | Precision |
| Decoder Models |  |  |  |  |  |  |  |  |  |  |  |  |
| Instruction Tuning |  |  |  |  |  |  |  |  |  |  |  |  |
| GPTNeox-3.6B | 0.578  (0.029) | 0.590  (0.043) | 0.650  (0.006) | 0.563  (0.028) | 0.577  (0.041) | 0.638  (0.006) | 0.557  (0.027) | 0.572  (0.041) | 0.633  (0.006) | 0.549  (0.027) | 0.566  (0.040) | 0.627  (0.006) |
| LLM-jp-13B v1.0 | 0.632  (0.014) | 0.614  (0.021) | 0.743  (0.006) | 0.620  (0.014) | 0.603  (0.021) | 0.737  (0.007) | 0.616  (0.014) | 0.599  (0.021) | 0.735  (0.007) | 0.610  (0.014) | 0.594  (0.021) | 0.732  (0.007) |
| Swallow-7B | 0.663  (0.011) | 0.676  (0.035) | 0.730  (0.019) | 0.649  (0.009) | 0.664  (0.034) | 0.721  (0.022) | 0.643  (0.008) | 0.660  (0.034) | 0.717  (0.023) | 0.636  (0.007) | 0.655  (0.035) | 0.714  (0.024) |
| Swallow-13B | 0.674  (0.015) | 0.680  (0.026) | 0.747  (0.009) | 0.662  (0.015) | 0.667  (0.025) | 0.740  (0.010) | 0.657  (0.015) | 0.663  (0.025) | 0.738  (0.010) | 0.651  (0.015) | 0.657  (0.024) | 0.735  (0.011) |
| Swallow-70B | 0.683  (0.025) | 0.691  (0.037) | **0.751**  **(0.013)** | 0.670  (0.024) | 0.679  (0.036) | **0.743**  **(0.014)** | 0.665  (0.023) | 0.675  (0.036) | 0.741  (0.014) | 0.658  (0.023) | 0.670  (0.035) | 0.737  (0.014) |
| Swallow-MS-7B | 0.654  (0.028) | 0.656  (0.046) | 0.734  (0.015) | 0.641  (0.027) | 0.645  (0.044) | 0.726  (0.016) | 0.637  (0.026) | 0.641  (0.044) | 0.724  (0.016) | 0.631  (0.026) | 0.635  (0.043) | 0.721  (0.016) |
| Token Classification |  |  |  |  |  |  |  |  |  |  |  |  |
| GPTNeox-3.6B | 0.701  (0.005) | 0.740  (0.014) | 0.711  (0.007) | 0.676  (0.005) | 0.699  (0.014) | 0.707  (0.007) | 0.667  (0.005) | 0.686  (0.014) | 0.706  (0.007) | 0.655  (0.005) | 0.669  (0.014) | 0.704  (0.007) |
| LLM-jp-13B v1.0 | 0.708  (0.004) | 0.752  (0.017) | 0.715  (0.010) | 0.684  (0.004) | 0.713  (0.017) | 0.711  (0.010) | 0.675  (0.004) | 0.700  (0.017) | 0.710  (0.010) | 0.664  (0.004) | 0.685  (0.017) | 0.708  (0.010) |
| Swallow-7B | 0.709  (0.003) | 0.744  (0.010) | 0.724  (0.008) | 0.684  (0.004) | 0.704  (0.012) | 0.720  (0.009) | 0.676  (0.004) | 0.691  (0.013) | 0.719  (0.009) | 0.664  (0.004) | 0.675  (0.013) | 0.717  (0.009) |
| Swallow-13B | 0.712  (0.003) | 0.750  (0.014) | 0.724  (0.008) | 0.689  (0.003) | 0.714  (0.014) | 0.719  (0.008) | 0.681  (0.003) | 0.703  (0.014) | 0.718  (0.008) | 0.670  (0.003) | 0.688  (0.013) | 0.715  (0.008) |
| Swallow-70B | 0.716  (0.006) | 0.754  (0.014) | 0.727  (0.002) | 0.692  (0.007) | 0.716  (0.016) | 0.724  (0.002) | 0.684  (0.008) | 0.704  (0.016) | 0.723  (0.002) | 0.673  (0.008) | 0.688  (0.017) | 0.722  (0.003) |
| Swallow-MS-7B | 0.714  (0.002) | 0.766  (0.011) | 0.712  (0.008) | 0.690  (0.002) | 0.727  (0.010) | 0.708  (0.008) | 0.681  (0.002) | 0.714  (0.010) | 0.706  (0.008) | 0.670  (0.002) | 0.698  (0.010) | 0.704  (0.008) |
| Encoder Models |  |  |  |  |  |  |  |  |  |  |  |  |
| Token Classification |  |  |  |  |  |  |  |  |  |  |  |  |
| DeBERTaV2-base | 0.736  (0.006) | 0.776  (0.014) | 0.745  (0.007) | 0.718  (0.007) | 0.749  (0.015) | 0.742  (0.007) | 0.712  (0.007) | 0.740  (0.016) | 0.741  (0.007) | 0.704  (0.008) | 0.729  (0.017) | 0.740  (0.007) |
| RoBERTa-base | 0.728  (0.004) | 0.762  (0.015) | 0.744  (0.008) | 0.709  (0.004) | 0.733  (0.014) | 0.741  (0.009) | 0.703  (0.004) | 0.724  (0.013) | 0.740  (0.009) | 0.693  (0.003) | 0.711  (0.013) | 0.739  (0.008) |
| JMedRoBERTa | 0.736  (0.003) | **0.783**  **(0.014)** | 0.739  (0.010) | 0.716  (0.003) | **0.753**  **(0.014)** | 0.734  (0.010) | 0.708  (0.003) | **0.744**  **(0.014)** | 0.732  (0.011) | 0.699  (0.003) | 0.731  (0.014) | 0.729  (0.011) |
| JMedDeBERTa(s) | **0.738**  **(0.003)** | 0.781  (0.012) | 0.744  (0.009) | **0.720**  **(0.003)** | **0.753**  **(0.012)** | 0.741  (0.010) | **0.714**  **(0.003)** | **0.744**  **(0.012)** | 0.740  (0.010) | **0.705**  **(0.003)** | **0.733**  **(0.012)** | 0.739  (0.010) |
| JMedDeBERTa(c) | 0.737  (0.005) | 0.778  (0.014) | 0.745  (0.009) | 0.719  (0.005) | 0.750  (0.014) | **0.743**  **(0.009)** | 0.713  (0.005) | 0.741  (0.014) | **0.742**  **(0.009)** | 0.704  (0.005) | 0.729  (0.014) | **0.741**  **(0.009)** |

Table S5. Case-level Marker Matching Score for 17 encoder/decoder models on the J-CaseMap test set. In the marker matching score, each clinical concept is counted as 1 unit regardless of span length. Marker-level F1, Recall, and Precision were calculated for each case report and averaged. Results are shown for 4 conditions of p = 1, 1.5, 2, 100. Values are presented as mean (SD).

| J-CaseMap  Each Extraction Targets | p=1 | | | p=1.5 | | | p=2 | | | p=100 | | |
| --- | --- | --- | --- | --- | --- | --- | --- | --- | --- | --- | --- | --- |
|  | F1 | Recall | Precision | F1 | Recall | Precision | F1 | Recall | Precision | F1 | Recall | Precision |
| Decoder Models |  |  |  |  |  |  |  |  |  |  |  |  |
| Instruction Tuning |  |  |  |  |  |  |  |  |  |  |  |  |
| GPTNeox-3.6B | 0.527  (0.025) | 0.623  (0.042) | 0.499  (0.010) | 0.522  (0.024) | 0.618  (0.041) | 0.495  (0.010) | 0.520  (0.024) | 0.616  (0.041) | 0.494  (0.010) | 0.518  (0.024) | 0.614  (0.041) | 0.492  (0.010) |
| LLM-jp-13B v1.0 | 0.655  (0.012) | 0.642  (0.018) | 0.739  (0.006) | 0.652  (0.012) | 0.638  (0.018) | 0.737  (0.006) | 0.650  (0.012) | 0.636  (0.018) | 0.736  (0.006) | 0.648  (0.012) | 0.634  (0.018) | 0.735  (0.006) |
| Swallow-7B | 0.688  (0.010) | 0.701  (0.028) | 0.735  (0.012) | 0.684  (0.010) | 0.696  (0.028) | 0.732  (0.012) | 0.682  (0.010) | 0.694  (0.028) | 0.731  (0.013) | 0.680  (0.010) | 0.692  (0.028) | 0.730  (0.013) |
| Swallow-13B | 0.698  (0.015) | 0.710  (0.030) | 0.743  (0.008) | 0.693  (0.015) | 0.705  (0.029) | 0.740  (0.009) | 0.692  (0.015) | 0.704  (0.029) | 0.740  (0.009) | 0.690  (0.015) | 0.701  (0.029) | 0.739  (0.009) |
| Swallow-70B | 0.705  (0.026) | 0.717  (0.040) | **0.750**  **(0.009)** | 0.700  (0.026) | 0.713  (0.039) | **0.747**  **(0.010)** | 0.698  (0.026) | 0.711  (0.039) | **0.746**  **(0.010)** | 0.696  (0.026) | 0.708  (0.039) | **0.745**  **(0.010)** |
| Swallow-MS-7B | 0.675  (0.028) | 0.679  (0.048) | 0.732  (0.012) | 0.670  (0.028) | 0.675  (0.048) | 0.730  (0.012) | 0.669  (0.028) | 0.673  (0.048) | 0.729  (0.012) | 0.666  (0.028) | 0.671  (0.047) | 0.728  (0.012) |
| Token Classification |  |  |  |  |  |  |  |  |  |  |  |  |
| GPTNeox-3.6B | 0.652  (0.004) | 0.780  (0.013) | 0.594  (0.007) | 0.643  (0.004) | 0.760  (0.013) | 0.593  (0.007) | 0.640  (0.005) | 0.753  (0.013) | 0.593  (0.007) | 0.636  (0.005) | 0.745  (0.013) | 0.593  (0.007) |
| LLM-jp-13B v1.0 | 0.657  (0.002) | 0.795  (0.017) | 0.594  (0.010) | 0.649  (0.002) | 0.777  (0.017) | 0.594  (0.010) | 0.646  (0.002) | 0.770  (0.018) | 0.593  (0.010) | 0.642  (0.002) | 0.762  (0.018) | 0.593  (0.010) |
| Swallow-7B | 0.660  (0.004) | 0.784  (0.008) | 0.606  (0.010) | 0.652  (0.003) | 0.765  (0.009) | 0.605  (0.011) | 0.649  (0.003) | 0.758  (0.009) | 0.604  (0.011) | 0.645  (0.003) | 0.750  (0.009) | 0.604  (0.011) |
| Swallow-13B | 0.660  (0.002) | 0.788  (0.014) | 0.604  (0.009) | 0.652  (0.003) | 0.771  (0.014) | 0.603  (0.010) | 0.650  (0.003) | 0.765  (0.015) | 0.603  (0.010) | 0.646  (0.003) | 0.758  (0.015) | 0.602  (0.010) |
| Swallow-70B | 0.668  (0.005) | 0.796  (0.015) | 0.611  (0.004) | 0.660  (0.005) | 0.778  (0.015) | 0.610  (0.004) | 0.657  (0.005) | 0.772  (0.016) | 0.610  (0.004) | 0.653  (0.006) | 0.764  (0.016) | 0.609  (0.004) |
| Swallow-MS-7B | 0.658  (0.003) | 0.804  (0.013) | 0.591  (0.008) | 0.650  (0.003) | 0.785  (0.013) | 0.590  (0.008) | 0.647  (0.003) | 0.779  (0.013) | 0.589  (0.008) | 0.643  (0.003) | 0.771  (0.013) | 0.589  (0.008) |
| Encoder Models |  |  |  |  |  |  |  |  |  |  |  |  |
| Token Classification |  |  |  |  |  |  |  |  |  |  |  |  |
| DeBERTaV2-base | **0.725**  **(0.008)** | 0.820  (0.013) | 0.685  (0.016) | **0.720**  **(0.009)** | 0.808  (0.013) | 0.684  (0.016) | **0.718**  **(0.009)** | 0.805  (0.013) | 0.683  (0.016) | **0.715**  **(0.009)** | 0.799  (0.014) | 0.683  (0.016) |
| RoBERTa-base | 0.718  (0.004) | 0.805  (0.018) | 0.684  (0.013) | 0.712  (0.004) | 0.793  (0.017) | 0.683  (0.013) | 0.710  (0.004) | 0.789  (0.017) | 0.682  (0.013) | 0.707  (0.004) | 0.783  (0.017) | 0.682  (0.013) |
| JMedRoBERTa | 0.724  (0.002) | 0.824  (0.013) | 0.679  (0.011) | 0.717  (0.002) | 0.812  (0.013) | 0.678  (0.012) | 0.715  (0.002) | 0.808  (0.014) | 0.677  (0.012) | 0.711  (0.002) | 0.802  (0.014) | 0.676  (0.012) |
| JMedDeBERTa(s) | 0.722  (0.004) | **0.827**  **(0.010)** | 0.675  (0.010) | 0.716  (0.004) | **0.815**  **(0.010)** | 0.674  (0.011) | 0.714  (0.004) | **0.811**  **(0.010)** | 0.674  (0.011) | 0.711  (0.004) | **0.805**  **(0.010)** | 0.674  (0.011) |
| JMedDeBERTa(c) | 0.716  (0.005) | 0.821  (0.015) | 0.668  (0.013) | 0.710  (0.005) | 0.809  (0.015) | 0.668  (0.013) | 0.707  (0.005) | 0.805  (0.015) | 0.668  (0.013) | 0.705  (0.005) | 0.799  (0.015) | 0.667  (0.013) |

Table S6. Case-level Character Segment Score for 17 encoder/decoder models on the J-CaseMap test set. Character-level F1, Recall, and Precision were calculated for each case report and averaged. Values are presented as mean (SD).

| J-CaseMap  Each Extraction Targets | F1 | Recall | Precision |
| --- | --- | --- | --- |
|  |  |  |  |
| Decoder Models |  |  |  |
| Instruction Tuning |  |  |  |
| GPTNeox-3.6B | 0.578  (0.029) | 0.590  (0.043) | 0.650  (0.006) |
| LLM-jp-13B v1.0 | 0.632  (0.014) | 0.614  (0.021) | 0.743  (0.006) |
| Swallow-7B | 0.663  (0.011) | 0.676  (0.035) | 0.730  (0.019) |
| Swallow-13B | 0.674  (0.015) | 0.680  (0.026) | 0.747  (0.009) |
| Swallow-70B | 0.683  (0.025) | 0.691  (0.037) | **0.751**  **(0.013)** |
| Swallow-MS-7B | 0.654  (0.028) | 0.656  (0.046) | 0.734  (0.015) |
| Token Classification |  |  |  |
| GPTNeox-3.6B | 0.701  (0.005) | 0.740  (0.014) | 0.711  (0.007) |
| LLM-jp-13B v1.0 | 0.708  (0.004) | 0.752  (0.017) | 0.715  (0.010) |
| Swallow-7B | 0.709  (0.003) | 0.744  (0.010) | 0.724  (0.008) |
| Swallow-13B | 0.712  (0.003) | 0.750  (0.014) | 0.724  (0.008) |
| Swallow-70B | 0.716  (0.006) | 0.754  (0.014) | 0.727  (0.002) |
| Swallow-MS-7B | 0.714  (0.002) | 0.766  (0.011) | 0.712  (0.008) |
| Encoder Models |  |  |  |
| Token Classification |  |  |  |
| DeBERTaV2-base | 0.736  (0.006) | 0.776  (0.014) | 0.745  (0.007) |
| RoBERTa-base | 0.728  (0.004) | 0.762  (0.015) | 0.744  (0.008) |
| JMedRoBERTa | 0.736  (0.003) | **0.783**  **(0.014)** | 0.739  (0.010) |
| JMedDeBERTa(s) | **0.738**  **(0.003)** | 0.781  (0.012) | 0.744  (0.009) |
| JMedDeBERTa(c) | 0.737  (0.005) | 0.778  (0.014) | 0.745  (0.009) |

Table S7. Case-level Token Segment Score for 17 encoder/decoder models on the J-CaseMap test set. Token-level F1, Recall, and Precision were calculated for each case report and averaged. Values are presented as mean (SD).

| J-CaseMap  Each Extraction Targets | F1 | Recall | Precision |
| --- | --- | --- | --- |
|  |  |  |  |
| Decoder Models |  |  |  |
| Instruction Tuning |  |  |  |
| GPTNeox-3.6B | 0.586  (0.029) | 0.601  (0.043) | 0.645  (0.007) |
| LLM-jp-13B v1.0 | 0.633  (0.015) | 0.617  (0.021) | 0.743  (0.006) |
| Swallow-7B | 0.667  (0.011) | 0.681  (0.034) | 0.732  (0.018) |
| Swallow-13B | 0.679  (0.015) | 0.684  (0.026) | 0.749  (0.009) |
| Swallow-70B | 0.687  (0.025) | 0.696  (0.037) | 0.753  (0.013) |
| Swallow-MS-7B | 0.658  (0.029) | 0.660  (0.046) | 0.735  (0.015) |
| Token Classification |  |  |  |
| GPTNeox-3.6B | 0.708  (0.005) | 0.724  (0.013) | 0.738  (0.006) |
| LLM-jp-13B v1.0 | 0.710  (0.006) | 0.730  (0.020) | 0.740  (0.011) |
| Swallow-7B | 0.712  (0.003) | 0.723  (0.010) | 0.750  (0.008) |
| Swallow-13B | 0.714  (0.005) | 0.731  (0.015) | 0.746  (0.009) |
| Swallow-70B | 0.720  (0.007) | 0.737  (0.015) | 0.753  (0.004) |
| Swallow-MS-7B | 0.714  (0.003) | 0.746  (0.012) | 0.731  (0.007) |
| Encoder Models |  |  |  |
| Token Classification |  |  |  |
| DeBERTaV2-base | 0.747  (0.006) | 0.781  (0.015) | 0.761  (0.005) |
| RoBERTa-base | 0.739  (0.004) | 0.764  (0.016) | 0.760  (0.009) |
| JMedRoBERTa | 0.732  (0.003) | 0.769  (0.014) | 0.749  (0.010) |
| JMedDeBERTa(s) | 0.733  (0.003) | 0.764  (0.011) | 0.756  (0.009) |
| JMedDeBERTa(c) | **0.749**  **(0.005)** | **0.782**  **(0.016)** | **0.764**  **(0.008)** |

Table S8. Hyperparameters used for pretraining and continual pretraining of JMedDeBERTa(s) and JMedDeBERTa(c). Both models were trained on 0.56 GB of Japanese medical text. Settings include maximum epochs, optimizer, effective global batch size, max token length, and DeepSpeed configuration.

| Hyperparameter | JMedDeBERTa(s),(c) |
| --- | --- |
|  |  |
| Max Training Epochs | 60 |
| Weight Decay | 0.01 |
| Learning Rate | 2.00E-04 |
| Effective Global Batch Size | 2048 |
| Max Token Length | 512 |
| Optimizer | AdamW |
| Warmup Steps | 3000 |
| DeepSpeed | Zero2 |

Figure S1. Training and validation loss curves during pretraining of JmedDeBERTa(s)

To monitor training behavior, we tracked JmedDeBERTa(s) loss on both the training and validation splits throughout pretraining. Both losses decrease smoothly and then plateau. By ~80K update steps, the training loss decreased from ~5.9 to ~1.55, and the validation loss from ~3.35 to ~1.60.


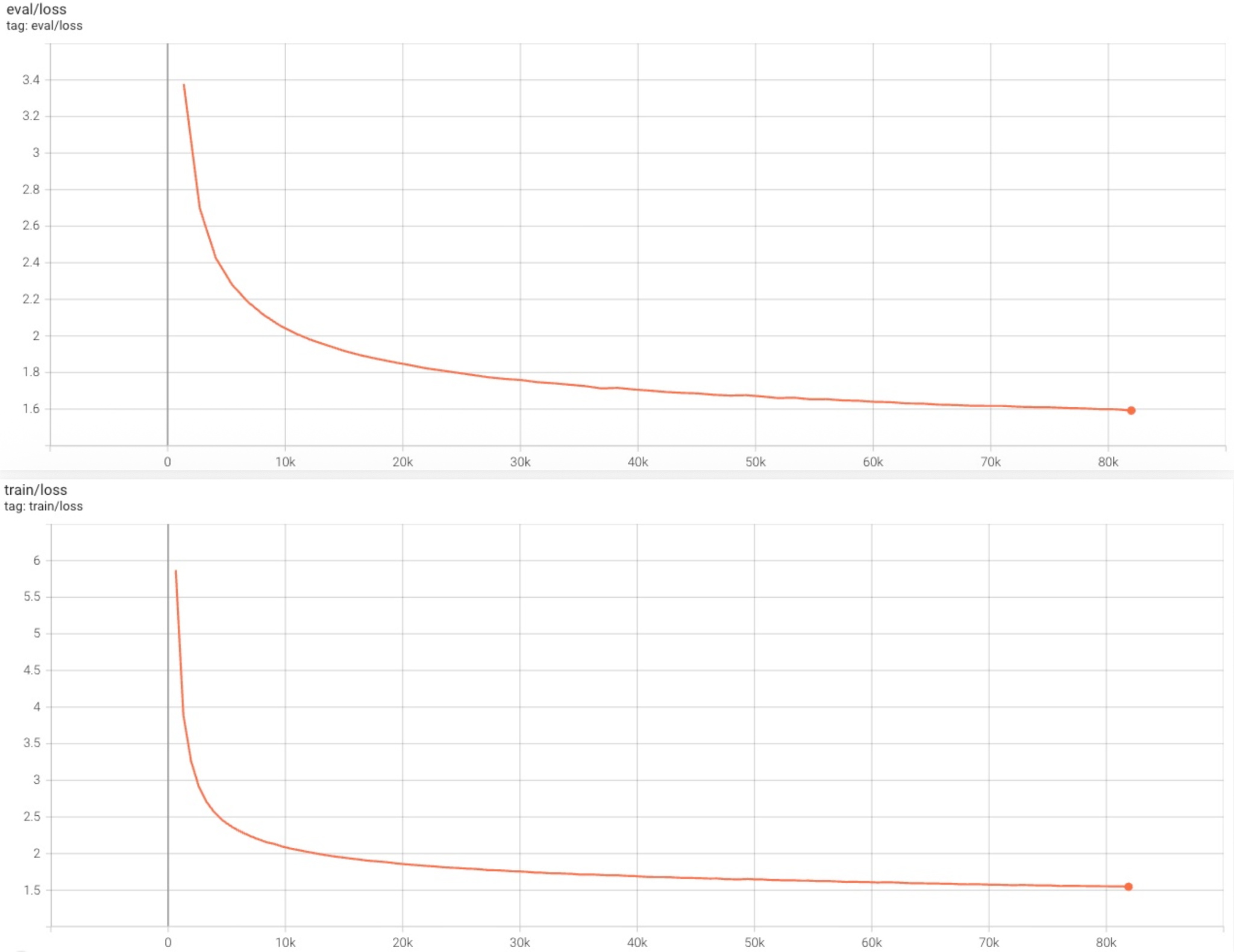


Figure S2. Training and validation loss curves during pretraining of JmedDeBERTa(c)

To monitor training behavior, we tracked JmedDeBERTa(c) loss on both the training and validation splits throughout additional pretraining. Both losses decrease smoothly and then plateau. By ~80K update steps, the training loss decreased from ~1.5 to ~0.65, and the validation loss from ~1.05 to ~0.65.


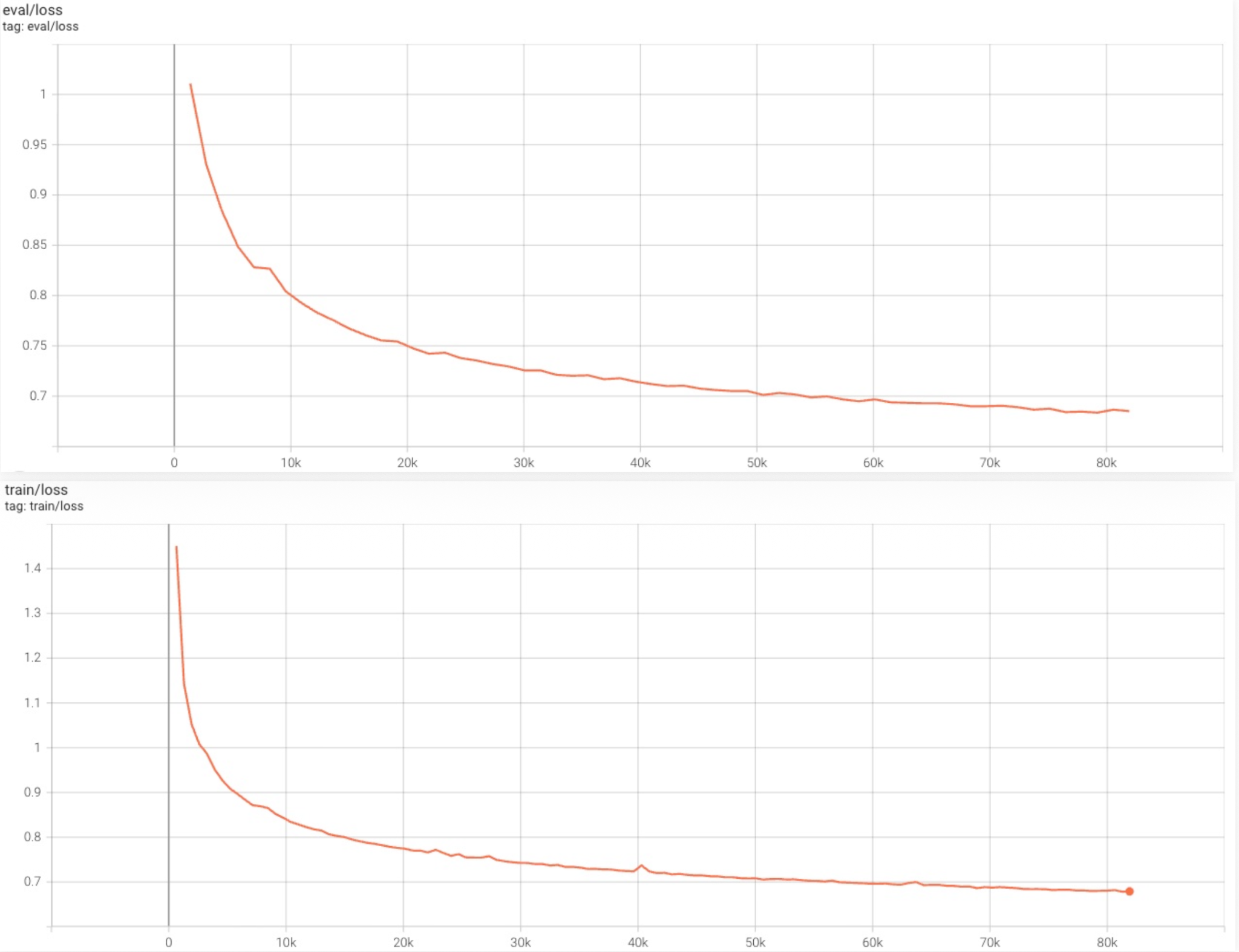


Table S9. Fine-tuning hyperparameters for Decoder and Encoder models. Decoder models (LLM-jp-13B v1.0, GPT-NeoX-3.6B, Swallow-7B/13B/70B, Swallow-MS-7B) were fine-tuned via instruction tuning or token classification using low-rank adaptation (LoRA). Encoder models (RoBERTa-base, DeBERTaV2-base, JMedRoBERTa, JMedDeBERTa(s), JMedDeBERTa(c)) were fine-tuned via token classification only. The table lists learning rate, batch size, maximum sequence length, number of epochs, and LoRA settings.

| Hyperparameter | Decoder Models | Encoder Models |
| --- | --- | --- |
|  |  |  |
| Max Training Epochs | 10 | 10 |
| Weight Decay | 0.01 | 0.01 |
| Learning Rate | 1.00E-04 | 3.00E-05 |
| Batch Size | 64 | 32 |
| Max Token Length |  |  |
| Token Classification | 768 | 512 |
| Instruction Tuning | 1792 |  |
| Lora rank | 12 |  |
| Lora alpha | 32 |  |
| Lora dropout | 0.1 |  |
| Target Module |  |  |
| GPT-NeoX-3.6B | query_key_value |  |
| LLM-jp-13B v1.0 | c_attn |  |
| Swallow-7B,13B,70B | q_proj, v_proj |  |
| Swallow-MS-7B | q_proj, v_proj |  |

Table S10. Performance of all 17 comparison models in terms of Weighted Soft Matching Score on the public dataset MRNER-disease. All extraction targets are evaluated collectively. F1, Recall, and Precision are reported for each model under 4 conditions of the fragmentation penalty parameter p (1, 1.5, 2, 100). Values are presented as mean (SD). This task is a conventional clinical NER task targeting actual disease names observed in patients.

| MRNER-disease  All Extraction Targets | p=1 | | | p=1.5 | | | p=2 | | | p=100 | | |
| --- | --- | --- | --- | --- | --- | --- | --- | --- | --- | --- | --- | --- |
|  | F1 | Recall | Precision | F1 | Recall | Precision | F1 | Recall | Precision | F1 | Recall | Precision |
| Decoder Models |  |  |  |  |  |  |  |  |  |  |  |  |
| Instruction Tuning |  |  |  |  |  |  |  |  |  |  |  |  |
| GPTNeox-3.6B | 0.373  (0.067) | 0.286  (0.066) | 0.560  (0.116) | 0.369  (0.065) | 0.281  (0.063) | 0.560  (0.116) | 0.368  (0.064) | 0.279  (0.062) | 0.560  (0.116) | 0.365  (0.063) | 0.276  (0.061) | 0.560  (0.116) |
| LLM-jp-13B v1.0 | 0.585  (0.071) | 0.518  (0.099) | 0.680  (0.050) | 0.581  (0.071) | 0.514  (0.099) | 0.675  (0.053) | 0.579  (0.072) | 0.512  (0.099) | 0.673  (0.054) | 0.576  (0.072) | 0.510  (0.099) | 0.671  (0.055) |
| Swallow-7B | 0.597  (0.046) | 0.557  (0.088) | 0.657  (0.084) | 0.591  (0.045) | 0.551  (0.088) | 0.652  (0.083) | 0.588  (0.044) | 0.548  (0.088) | 0.650  (0.082) | 0.585  (0.044) | 0.544  (0.087) | 0.646  (0.081) |
| Swallow-13B | 0.650  (0.051) | 0.600  (0.096) | 0.722  (0.075) | 0.644  (0.050) | 0.593  (0.096) | 0.718  (0.076) | 0.641  (0.049) | 0.590  (0.096) | 0.716  (0.076) | 0.637  (0.049) | 0.585  (0.096) | 0.714  (0.076) |
| Swallow-70B | 0.660  (0.064) | 0.626  (0.125) | 0.719  (0.076) | 0.655  (0.063) | 0.621  (0.124) | 0.714  (0.077) | 0.653  (0.062) | 0.619  (0.124) | 0.713  (0.076) | 0.650  (0.062) | 0.615  (0.124) | 0.710  (0.076) |
| Swallow-MS-7B | 0.577  (0.054) | 0.539  (0.079) | 0.626  (0.057) | 0.571  (0.052) | 0.533  (0.079) | 0.622  (0.055) | 0.569  (0.052) | 0.531  (0.080) | 0.621  (0.054) | 0.566  (0.052) | 0.527  (0.080) | 0.619  (0.054) |
| Token Classification |  |  |  |  |  |  |  |  |  |  |  |  |
| GPTNeox-3.6B | 0.663  (0.038) | 0.624  (0.070) | 0.713  (0.036) | 0.648  (0.036) | 0.599  (0.068) | 0.712  (0.036) | 0.642  (0.036) | 0.590  (0.068) | 0.711  (0.036) | 0.635  (0.036) | 0.578  (0.067) | 0.711  (0.036) |
| LLM-jp-13B v1.0 | 0.694  (0.029) | 0.665  (0.066) | 0.731  (0.040) | 0.676  (0.030) | 0.635  (0.067) | 0.729  (0.039) | 0.669  (0.031) | 0.624  (0.067) | 0.729  (0.039) | 0.660  (0.031) | 0.609  (0.067) | 0.728  (0.038) |
| Swallow-7B | 0.676  (0.033) | 0.645  (0.077) | 0.720  (0.051) | 0.656  (0.032) | 0.612  (0.076) | 0.719  (0.051) | 0.649  (0.032) | 0.600  (0.075) | 0.718  (0.051) | 0.639  (0.032) | 0.584  (0.075) | 0.717  (0.052) |
| Swallow-13B | 0.661  (0.022) | 0.616  (0.063) | 0.720  (0.036) | 0.639  (0.024) | 0.581  (0.064) | 0.719  (0.036) | 0.631  (0.025) | 0.568  (0.064) | 0.718  (0.036) | 0.620  (0.027) | 0.550  (0.065) | 0.717  (0.036) |
| Swallow-70B | 0.642  (0.030) | 0.604  (0.064) | 0.691  (0.016) | 0.620  (0.030) | 0.566  (0.062) | 0.689  (0.016) | 0.611  (0.031) | 0.552  (0.061) | 0.689  (0.016) | 0.599  (0.032) | 0.534  (0.062) | 0.688  (0.016) |
| Swallow-MS-7B | 0.632  (0.019) | 0.610  (0.041) | 0.657  (0.034) | 0.611  (0.019) | 0.574  (0.040) | 0.657  (0.034) | 0.604  (0.019) | 0.561  (0.040) | 0.657  (0.034) | 0.593  (0.021) | 0.543  (0.042) | 0.657  (0.034) |
| Encoder Models |  |  |  |  |  |  |  |  |  |  |  |  |
| Token Classification |  |  |  |  |  |  |  |  |  |  |  |  |
| DeBERTaV2-base | **0.794**  **(0.018)** | **0.795**  **(0.020)** | 0.795  (0.051) | **0.784**  **(0.017)** | **0.781**  **(0.023)** | 0.791  (0.050) | **0.781**  **(0.017)** | **0.775**  **(0.024)** | 0.789  (0.050) | **0.776**  **(0.017)** | **0.768**  **(0.026)** | 0.787  (0.049) |
| RoBERTa-base | 0.768  (0.007) | 0.759  (0.029) | 0.778  (0.022) | 0.757  (0.006) | 0.741  (0.026) | 0.773  (0.019) | 0.752  (0.006) | 0.735  (0.026) | 0.772  (0.018) | 0.747  (0.007) | 0.727  (0.025) | 0.769  (0.016) |
| JMedRoBERTa | 0.740  (0.015) | 0.711  (0.009) | 0.772  (0.031) | 0.727  (0.010) | 0.689  (0.011) | 0.771  (0.032) | 0.722  (0.008) | 0.680  (0.014) | 0.770  (0.033) | 0.715  (0.005) | 0.668  (0.018) | 0.769  (0.033) |
| JMedDeBERTa(s) | 0.788  (0.027) | 0.744  (0.024) | **0.838**  **(0.043)** | 0.777  (0.024) | 0.729  (0.022) | **0.834**  **(0.046)** | 0.773  (0.023) | 0.723  (0.024) | **0.832**  **(0.047)** | 0.767  (0.022) | 0.716  (0.027) | **0.829**  **(0.048)** |
| JMedDeBERTa(c) | 0.788  (0.016) | 0.787  (0.019) | 0.790  (0.040) | 0.779  (0.016) | 0.771  (0.016) | 0.788  (0.038) | 0.775  (0.016) | 0.765  (0.015) | 0.787  (0.038) | 0.769  (0.016) | 0.755  (0.013) | 0.785  (0.037) |

Table S11. Marker matching score performance of 17 encoder/decoder models on the public dataset MRNER-disease. Performance is calculated using the marker matching score, evaluating all extraction targets collectively. In this metric, each annotated disease name (marker span) is treated as 1 unit regardless of its length. F1, Recall, and Precision are shown for 4 conditions of the fragmentation penalty parameter p (1, 1.5, 2, 100). Values are presented as mean (SD).

| MRNER-disease  All Extraction Targets | p=1 | | | p=1.5 | | | p=2 | | | p=100 | | |
| --- | --- | --- | --- | --- | --- | --- | --- | --- | --- | --- | --- | --- |
|  | F1 | Recall | Precision | F1 | Recall | Precision | F1 | Recall | Precision | F1 | Recall | Precision |
| Decoder Models |  |  |  |  |  |  |  |  |  |  |  |  |
| Instruction Tuning |  |  |  |  |  |  |  |  |  |  |  |  |
| GPTNeox-3.6B | 0.414  (0.075) | 0.325  (0.080) | 0.595  (0.103) | 0.412  (0.074) | 0.323  (0.079) | 0.595  (0.103) | 0.411  (0.074) | 0.322  (0.078) | 0.595  (0.103) | 0.410  (0.073) | 0.320  (0.078) | 0.595  (0.103) |
| LLM-jp-13B v1.0 | 0.640  (0.082) | 0.549  (0.115) | 0.779  (0.053) | 0.637  (0.082) | 0.547  (0.115) | 0.777  (0.053) | 0.637  (0.082) | 0.546  (0.115) | 0.776  (0.053) | 0.635  (0.082) | 0.545  (0.115) | 0.776  (0.052) |
| Swallow-7B | 0.641  (0.052) | 0.590  (0.091) | 0.714  (0.086) | 0.638  (0.052) | 0.588  (0.091) | 0.713  (0.086) | 0.638  (0.052) | 0.586  (0.091) | 0.712  (0.086) | 0.636  (0.051) | 0.585  (0.090) | 0.711  (0.086) |
| Swallow-13B | 0.684  (0.049) | 0.625  (0.091) | 0.769  (0.083) | 0.681  (0.049) | 0.622  (0.091) | 0.768  (0.084) | 0.680  (0.049) | 0.620  (0.091) | 0.767  (0.084) | 0.679  (0.049) | 0.618  (0.091) | 0.767  (0.084) |
| Swallow-70B | 0.713  (0.048) | 0.668  (0.102) | 0.784  (0.084) | 0.711  (0.047) | 0.665  (0.102) | 0.782  (0.084) | 0.710  (0.047) | 0.664  (0.101) | 0.782  (0.084) | 0.709  (0.047) | 0.662  (0.101) | 0.781  (0.083) |
| Swallow-MS-7B | 0.630  (0.070) | 0.578  (0.087) | 0.697  (0.074) | 0.628  (0.070) | 0.575  (0.087) | 0.696  (0.073) | 0.627  (0.070) | 0.574  (0.087) | 0.695  (0.073) | 0.626  (0.070) | 0.573  (0.087) | 0.694  (0.073) |
| Token Classification |  |  |  |  |  |  |  |  |  |  |  |  |
| GPTNeox-3.6B | 0.636  (0.036) | 0.647  (0.082) | 0.631  (0.023) | 0.630  (0.036) | 0.634  (0.081) | 0.631  (0.023) | 0.627  (0.037) | 0.629  (0.081) | 0.631  (0.023) | 0.624  (0.037) | 0.623  (0.081) | 0.631  (0.023) |
| LLM-jp-13B v1.0 | 0.666  (0.021) | 0.691  (0.072) | 0.649  (0.030) | 0.659  (0.022) | 0.676  (0.074) | 0.648  (0.030) | 0.656  (0.023) | 0.670  (0.074) | 0.648  (0.030) | 0.651  (0.024) | 0.662  (0.076) | 0.648  (0.030) |
| Swallow-7B | 0.646  (0.040) | 0.668  (0.084) | 0.634  (0.053) | 0.638  (0.039) | 0.651  (0.082) | 0.633  (0.053) | 0.634  (0.039) | 0.645  (0.081) | 0.633  (0.053) | 0.630  (0.038) | 0.636  (0.080) | 0.633  (0.053) |
| Swallow-13B | 0.639  (0.029) | 0.636  (0.052) | 0.645  (0.040) | 0.629  (0.029) | 0.618  (0.053) | 0.645  (0.040) | 0.626  (0.029) | 0.611  (0.054) | 0.644  (0.040) | 0.620  (0.030) | 0.601  (0.055) | 0.644  (0.040) |
| Swallow-70B | 0.612  (0.028) | 0.624  (0.064) | 0.603  (0.026) | 0.602  (0.028) | 0.605  (0.063) | 0.603  (0.026) | 0.598  (0.028) | 0.598  (0.063) | 0.603  (0.026) | 0.593  (0.029) | 0.588  (0.064) | 0.602  (0.025) |
| Swallow-MS-7B | 0.606  (0.024) | 0.648  (0.042) | 0.573  (0.041) | 0.597  (0.024) | 0.627  (0.042) | 0.573  (0.040) | 0.594  (0.024) | 0.620  (0.043) | 0.573  (0.040) | 0.588  (0.024) | 0.609  (0.044) | 0.573  (0.040) |
| Encoder Models |  |  |  |  |  |  |  |  |  |  |  |  |
| Token Classification |  |  |  |  |  |  |  |  |  |  |  |  |
| DeBERTaV2-base | 0.785  (0.030) | 0.812  (0.014) | 0.762  (0.060) | 0.782  (0.029) | **0.806**  **(0.015)** | 0.761  (0.059) | **0.781**  **(0.029)** | **0.804**  **(0.016)** | 0.761  (0.059) | 0.779  (0.028) | **0.800**  **(0.016)** | 0.761  (0.059) |
| RoBERTa-base | 0.763  (0.021) | 0.780  (0.040) | 0.748  (0.028) | 0.758  (0.023) | 0.772  (0.041) | 0.747  (0.028) | 0.757  (0.023) | 0.769  (0.041) | 0.746  (0.027) | 0.754  (0.024) | 0.765  (0.042) | 0.745  (0.027) |
| JMedRoBERTa | 0.754  (0.025) | 0.761  (0.031) | 0.748  (0.024) | 0.748  (0.025) | 0.750  (0.030) | 0.747  (0.025) | 0.746  (0.024) | 0.746  (0.030) | 0.747  (0.025) | 0.743  (0.024) | 0.740  (0.030) | 0.747  (0.025) |
| JMedDeBERTa(s) | **0.800**  **(0.022)** | 0.787  (0.022) | **0.814**  **(0.029)** | **0.796**  **(0.021)** | 0.780  (0.023) | **0.813**  **(0.029)** | 0.794  (0.021) | 0.777  (0.023) | **0.812**  **(0.030)** | **0.792**  **(0.020)** | 0.774  (0.024) | **0.811**  **(0.030)** |
| JMedDeBERTa(c) | 0.783  (0.018) | **0.814**  **(0.014)** | 0.755  (0.038) | 0.779  (0.018) | **0.806**  **(0.014)** | 0.754  (0.038) | 0.777  (0.018) | 0.803  (0.014) | 0.754  (0.037) | 0.775  (0.019) | 0.799  (0.013) | 0.753  (0.037) |

Table S12. Character-level performance (character segment score) of 17 encoder/decoder models on the public dataset MRNER-disease. Model performance is calculated on a character-by-character basis. F1, Recall, and Precision are shown. Values are presented as mean (SD).

| MRNER-disease  All Extraction Targets | F1 | Recall | Precision |
| --- | --- | --- | --- |
|  |  |  |  |
| Decoder Models |  |  |  |
| Instruction Tuning |  |  |  |
| GPTNeox-3.6B | 0.373  (0.067) | 0.286  (0.066) | 0.560  (0.116) |
| LLM-jp-13B v1.0 | 0.585  (0.071) | 0.518  (0.099) | 0.680  (0.050) |
| Swallow-7B | 0.597  (0.046) | 0.557  (0.088) | 0.657  (0.084) |
| Swallow-13B | 0.650  (0.051) | 0.600  (0.096) | 0.722  (0.075) |
| Swallow-70B | 0.660  (0.064) | 0.626  (0.125) | 0.719  (0.076) |
| Swallow-MS-7B | 0.577  (0.054) | 0.539  (0.079) | 0.626  (0.057) |
| Token Classification |  |  |  |
| GPTNeox-3.6B | 0.663  (0.038) | 0.624  (0.070) | 0.713  (0.036) |
| LLM-jp-13B v1.0 | 0.694  (0.029) | 0.665  (0.066) | 0.731  (0.040) |
| Swallow-7B | 0.676  (0.033) | 0.645  (0.077) | 0.720  (0.051) |
| Swallow-13B | 0.661  (0.022) | 0.616  (0.063) | 0.720  (0.036) |
| Swallow-70B | 0.642  (0.030) | 0.604  (0.064) | 0.691  (0.016) |
| Swallow-MS-7B | 0.632  (0.019) | 0.610  (0.041) | 0.657  (0.034) |
| Encoder Models |  |  |  |
| Token Classification |  |  |  |
| DeBERTaV2-base | **0.794**  **(0.018)** | **0.795**  **(0.020)** | 0.795  (0.051) |
| RoBERTa-base | 0.768  (0.007) | 0.759  (0.029) | 0.778  (0.022) |
| JMedRoBERTa | 0.740  (0.015) | 0.711  (0.009) | 0.772  (0.031) |
| JMedDeBERTa(s) | 0.788  (0.027) | 0.744  (0.024) | **0.838**  **(0.043)** |
| JMedDeBERTa(c) | 0.788  (0.016) | 0.787  (0.019) | 0.790  (0.040) |

Table S13. Token-level performance (token segment score) of 17 encoder/decoder models on the public dataset MRNER-disease. Model performance is calculated on a token-by-token basis. F1, Recall, and Precision are shown. Values are presented as mean (SD).

| MRNER-disease  All Extraction Targets | F1 | Recall | Precision |
| --- | --- | --- | --- |
|  |  |  |  |
| Decoder Models |  |  |  |
| Instruction Tuning |  |  |  |
| GPTNeox-3.6B | 0.388  (0.077) | 0.298  (0.076) | 0.578  (0.116) |
| LLM-jp-13B v1.0 | 0.601  (0.079) | 0.535  (0.108) | 0.692  (0.055) |
| Swallow-7B | 0.614  (0.048) | 0.581  (0.086) | 0.663  (0.089) |
| Swallow-13B | 0.668  (0.050) | 0.624  (0.099) | 0.730  (0.069) |
| Swallow-70B | 0.669  (0.059) | 0.637  (0.119) | 0.725  (0.077) |
| Swallow-MS-7B | 0.594  (0.065) | 0.558  (0.094) | 0.644  (0.074) |
| Token Classification |  |  |  |
| GPTNeox-3.6B | 0.682  (0.036) | 0.653  (0.070) | 0.719  (0.032) |
| LLM-jp-13B v1.0 | 0.708  (0.026) | 0.687  (0.066) | 0.736  (0.038) |
| Swallow-7B | 0.684  (0.032) | 0.658  (0.074) | 0.722  (0.053) |
| Swallow-13B | 0.675  (0.023) | 0.632  (0.063) | 0.730  (0.032) |
| Swallow-70B | 0.648  (0.027) | 0.609  (0.057) | 0.697  (0.022) |
| Swallow-MS-7B | 0.635  (0.018) | 0.624  (0.039) | 0.650  (0.039) |
| Encoder Models |  |  |  |
| Token Classification |  |  |  |
| DeBERTaV2-base | **0.810**  **(0.019)** | **0.819**  **(0.025)** | 0.805  (0.050) |
| RoBERTa-base | 0.796  (0.014) | 0.803  (0.023) | 0.790  (0.025) |
| JMedRoBERTa | 0.742  (0.017) | 0.726  (0.019) | 0.759  (0.025) |
| JMedDeBERTa(s) | 0.761  (0.027) | 0.712  (0.028) | **0.817**  **(0.043)** |
| JMedDeBERTa(c) | 0.807  (0.021) | 0.815  (0.022) | 0.802  (0.046) |

Table S14. Case-level Weighted Soft Matching Score for 17 encoder/decoder models on the public dataset MRNER-disease. F1, Recall, and Precision scores were calculated for all extraction targets (disease names) for each clinical document and averaged. Results are shown for 4 conditions of the fragmentation penalty parameter p (1, 1.5, 2, 100). Values are presented as mean (SD).

| MRNER-disease  Each Extraction Targets | p=1 | | | p=1.5 | | | p=2 | | | p=100 | | |
| --- | --- | --- | --- | --- | --- | --- | --- | --- | --- | --- | --- | --- |
|  | F1 | Recall | Precision | F1 | Recall | Precision | F1 | Recall | Precision | F1 | Recall | Precision |
| Decoder Models |  |  |  |  |  |  |  |  |  |  |  |  |
| Instruction Tuning |  |  |  |  |  |  |  |  |  |  |  |  |
| GPTNeox-3.6B | 0.326  (0.042) | 0.308  (0.038) | 0.440  (0.102) | 0.325  (0.041) | 0.305  (0.039) | 0.440  (0.102) | 0.324  (0.041) | 0.303  (0.040) | 0.440  (0.102) | 0.323  (0.041) | 0.302  (0.040) | 0.440  (0.102) |
| LLM-jp-13B v1.0 | 0.566  (0.092) | 0.540  (0.103) | 0.685  (0.099) | 0.563  (0.093) | 0.537  (0.105) | 0.682  (0.099) | 0.561  (0.093) | 0.535  (0.105) | 0.681  (0.099) | 0.559  (0.094) | 0.533  (0.106) | 0.680  (0.099) |
| Swallow-7B | 0.580  (0.060) | 0.607  (0.091) | 0.628  (0.044) | 0.575  (0.059) | 0.603  (0.091) | 0.624  (0.041) | 0.574  (0.058) | 0.601  (0.091) | 0.623  (0.041) | 0.571  (0.058) | 0.599  (0.091) | 0.621  (0.039) |
| Swallow-13B | 0.645  (0.065) | 0.644  (0.112) | 0.732  (0.078) | 0.640  (0.064) | 0.638  (0.111) | 0.729  (0.079) | 0.634  (0.064) | 0.632  (0.110) | 0.726  (0.079) | 0.634  (0.064) | 0.632  (0.110) | 0.726  (0.079) |
| Swallow-70B | 0.647  (0.056) | 0.645  (0.085) | 0.751  (0.078) | 0.643  (0.055) | 0.641  (0.084) | 0.748  (0.078) | 0.641  (0.055) | 0.640  (0.084) | 0.746  (0.078) | 0.639  (0.054) | 0.638  (0.084) | 0.745  (0.078) |
| Swallow-MS-7B | 0.577  (0.044) | 0.584  (0.089) | 0.676  (0.047) | 0.573  (0.044) | 0.578  (0.089) | 0.674  (0.045) | 0.571  (0.043) | 0.576  (0.090) | 0.673  (0.044) | 0.569  (0.043) | 0.573  (0.089) | 0.672  (0.043) |
| Token Classification |  |  |  |  |  |  |  |  |  |  |  |  |
| GPTNeox-3.6B | 0.642  (0.038) | 0.638  (0.069) | 0.716  (0.043) | 0.631  (0.037) | 0.617  (0.071) | 0.715  (0.043) | 0.626  (0.037) | 0.610  (0.072) | 0.715  (0.043) | 0.620  (0.038) | 0.601  (0.073) | 0.714  (0.043) |
| LLM-jp-13B v1.0 | 0.678  (0.035) | 0.669  (0.068) | 0.741  (0.036) | 0.661  (0.036) | 0.642  (0.069) | 0.739  (0.035) | 0.655  (0.036) | 0.632  (0.069) | 0.738  (0.034) | 0.645  (0.037) | 0.618  (0.069) | 0.737  (0.033) |
| Swallow-7B | 0.668  (0.031) | 0.662  (0.072) | 0.725  (0.065) | 0.651  (0.029) | 0.633  (0.073) | 0.725  (0.065) | 0.644  (0.028) | 0.622  (0.073) | 0.724  (0.065) | 0.635  (0.026) | 0.608  (0.072) | 0.724  (0.065) |
| Swallow-13B | 0.655  (0.022) | 0.639  (0.070) | 0.737  (0.034) | 0.636  (0.025) | 0.608  (0.073) | 0.736  (0.034) | 0.629  (0.026) | 0.597  (0.074) | 0.735  (0.033) | 0.618  (0.028) | 0.581  (0.076) | 0.734  (0.033) |
| Swallow-70B | 0.636  (0.032) | 0.629  (0.063) | 0.695  (0.027) | 0.618  (0.033) | 0.599  (0.063) | 0.694  (0.028) | 0.611  (0.033) | 0.588  (0.062) | 0.694  (0.028) | 0.601  (0.034) | 0.574  (0.063) | 0.694  (0.028) |
| Swallow-MS-7B | 0.619  (0.020) | 0.619  (0.036) | 0.670  (0.039) | 0.601  (0.021) | 0.586  (0.037) | 0.669  (0.038) | 0.593  (0.021) | 0.574  (0.038) | 0.669  (0.038) | 0.583  (0.022) | 0.558  (0.038) | 0.669  (0.038) |
| Encoder Models |  |  |  |  |  |  |  |  |  |  |  |  |
| Token Classification |  |  |  |  |  |  |  |  |  |  |  |  |
| DeBERTaV2-base | **0.793**  **(0.020)** | **0.808**  **(0.028)** | 0.812  (0.050) | **0.783**  **(0.016)** | **0.792**  **(0.032)** | 0.808  (0.049) | **0.779**  **(0.015)** | **0.786**  **(0.033)** | 0.807  (0.049) | **0.773**  **(0.014)** | **0.778**  **(0.036)** | 0.805  (0.048) |
| RoBERTa-base | 0.766  (0.017) | 0.775  (0.033) | 0.784  (0.018) | 0.754  (0.017) | 0.758  (0.032) | 0.781  (0.018) | 0.750  (0.017) | 0.752  (0.031) | 0.780  (0.018) | 0.743  (0.018) | 0.743  (0.031) | 0.778  (0.019) |
| JMedRoBERTa | 0.737  (0.014) | 0.727  (0.030) | 0.793  (0.037) | 0.725  (0.012) | 0.709  (0.036) | 0.792  (0.038) | 0.720  (0.012) | 0.702  (0.038) | 0.791  (0.039) | 0.713  (0.012) | 0.692  (0.041) | 0.790  (0.039) |
| JMedDeBERTa(s) | 0.775  (0.022) | 0.750  (0.006) | **0.848**  **(0.025)** | 0.765  (0.017) | 0.736  (0.015) | **0.844**  **(0.027)** | 0.760  (0.015) | 0.731  (0.018) | **0.843**  **(0.028)** | 0.754  (0.013) | 0.723  (0.024) | **0.840**  **(0.029)** |
| JMedDeBERTa(c) | 0.780  (0.019) | 0.791  (0.016) | 0.807  (0.024) | 0.770  (0.017) | 0.774  (0.017) | 0.805  (0.025) | 0.766  (0.017) | 0.768  (0.018) | 0.804  (0.025) | 0.760  (0.016) | 0.759  (0.019) | 0.803  (0.025) |

Table S15. Case-level Marker Matching Score for 17 encoder/decoder models on the public dataset MRNER-disease. In the marker matching score, each disease name is counted as 1 unit regardless of span length. Marker-level F1, Recall, and Precision were calculated for each clinical document and averaged. Results are shown for 4 conditions of p = 1, 1.5, 2, 100. Values are presented as mean (SD).

| MRNER-disease  Each Extraction Targets | p=1 | | | p=1.5 | | | p=2 | | | p=100 | | |
| --- | --- | --- | --- | --- | --- | --- | --- | --- | --- | --- | --- | --- |
|  | F1 | Recall | Precision | F1 | Recall | Precision | F1 | Recall | Precision | F1 | Recall | Precision |
| Decoder Models |  |  |  |  |  |  |  |  |  |  |  |  |
| Instruction Tuning |  |  |  |  |  |  |  |  |  |  |  |  |
| GPTNeox-3.6B | 0.347  (0.050) | 0.329  (0.033) | 0.449  (0.110) | 0.346  (0.049) | 0.327  (0.033) | 0.449  (0.110) | 0.346  (0.049) | 0.327  (0.034) | 0.449  (0.110) | 0.345  (0.049) | 0.325  (0.034) | 0.449  (0.110) |
| LLM-jp-13B v1.0 | 0.605  (0.095) | 0.561  (0.108) | 0.728  (0.108) | 0.604  (0.095) | 0.559  (0.108) | 0.726  (0.108) | 0.603  (0.095) | 0.558  (0.109) | 0.725  (0.108) | 0.602  (0.096) | 0.557  (0.109) | 0.725  (0.108) |
| Swallow-7B | 0.603  (0.068) | 0.619  (0.097) | 0.648  (0.060) | 0.600  (0.067) | 0.617  (0.097) | 0.646  (0.059) | 0.600  (0.067) | 0.617  (0.097) | 0.645  (0.059) | 0.598  (0.067) | 0.616  (0.097) | 0.644  (0.058) |
| Swallow-13B | 0.673  (0.067) | 0.669  (0.105) | 0.746  (0.090) | 0.670  (0.067) | 0.666  (0.105) | 0.744  (0.092) | 0.669  (0.068) | 0.665  (0.105) | 0.743  (0.092) | 0.667  (0.068) | 0.663  (0.105) | 0.742  (0.093) |
| Swallow-70B | 0.686  (0.060) | 0.673  (0.078) | 0.785  (0.076) | 0.684  (0.059) | 0.671  (0.078) | 0.783  (0.077) | 0.683  (0.059) | 0.670  (0.078) | 0.782  (0.077) | 0.682  (0.059) | 0.669  (0.078) | 0.781  (0.077) |
| Swallow-MS-7B | 0.615  (0.072) | 0.608  (0.092) | 0.707  (0.064) | 0.614  (0.072) | 0.606  (0.093) | 0.705  (0.063) | 0.613  (0.072) | 0.605  (0.093) | 0.705  (0.063) | 0.612  (0.072) | 0.603  (0.093) | 0.704  (0.062) |
| Token Classification |  |  |  |  |  |  |  |  |  |  |  |  |
| GPTNeox-3.6B | 0.611  (0.051) | 0.660  (0.079) | 0.636  (0.040) | 0.605  (0.051) | 0.648  (0.079) | 0.636  (0.040) | 0.602  (0.051) | 0.643  (0.080) | 0.636  (0.040) | 0.599  (0.052) | 0.637  (0.080) | 0.636  (0.040) |
| LLM-jp-13B v1.0 | 0.644  (0.035) | 0.689  (0.071) | 0.669  (0.036) | 0.635  (0.036) | 0.672  (0.071) | 0.668  (0.036) | 0.632  (0.037) | 0.666  (0.071) | 0.668  (0.035) | 0.627  (0.039) | 0.657  (0.072) | 0.667  (0.035) |
| Swallow-7B | 0.633  (0.042) | 0.682  (0.078) | 0.644  (0.068) | 0.624  (0.040) | 0.664  (0.078) | 0.644  (0.069) | 0.621  (0.039) | 0.658  (0.077) | 0.643  (0.069) | 0.616  (0.038) | 0.649  (0.077) | 0.643  (0.069) |
| Swallow-13B | 0.622  (0.032) | 0.659  (0.059) | 0.656  (0.058) | 0.613  (0.032) | 0.640  (0.059) | 0.656  (0.058) | 0.609  (0.032) | 0.633  (0.059) | 0.656  (0.058) | 0.604  (0.033) | 0.623  (0.060) | 0.655  (0.058) |
| Swallow-70B | 0.598  (0.034) | 0.652  (0.057) | 0.613  (0.027) | 0.589  (0.035) | 0.633  (0.056) | 0.613  (0.027) | 0.586  (0.035) | 0.626  (0.055) | 0.613  (0.027) | 0.581  (0.035) | 0.617  (0.055) | 0.613  (0.027) |
| Swallow-MS-7B | 0.588  (0.034) | 0.648  (0.031) | 0.588  (0.045) | 0.577  (0.034) | 0.625  (0.032) | 0.588  (0.045) | 0.573  (0.034) | 0.617  (0.032) | 0.588  (0.045) | 0.567  (0.034) | 0.605  (0.032) | 0.588  (0.044) |
| Encoder Models |  |  |  |  |  |  |  |  |  |  |  |  |
| Token Classification |  |  |  |  |  |  |  |  |  |  |  |  |
| DeBERTaV2-base | **0.788**  **(0.031)** | **0.820**  **(0.023)** | 0.790  (0.062) | **0.784**  **(0.029)** | **0.811**  **(0.022)** | 0.789  (0.061) | **0.782**  **(0.029)** | **0.808**  **(0.022)** | 0.789  (0.061) | **0.780**  **(0.028)** | **0.803**  **(0.022)** | 0.788  (0.061) |
| RoBERTa-base | 0.755  (0.031) | 0.794  (0.041) | 0.750  (0.026) | 0.750  (0.031) | 0.784  (0.040) | 0.748  (0.027) | 0.747  (0.032) | 0.780  (0.039) | 0.748  (0.027) | 0.743  (0.032) | 0.774  (0.039) | 0.747  (0.027) |
| JMedRoBERTa | 0.739  (0.024) | 0.761  (0.040) | 0.766  (0.042) | 0.733  (0.024) | 0.751  (0.041) | 0.766  (0.042) | 0.731  (0.024) | 0.746  (0.041) | 0.765  (0.042) | 0.727  (0.024) | 0.741  (0.042) | 0.765  (0.043) |
| JMedDeBERTa(s) | 0.781  (0.019) | 0.780  (0.015) | **0.824**  **(0.026)** | 0.776  (0.017) | 0.773  (0.018) | **0.823**  **(0.027)** | 0.774  (0.016) | 0.770  (0.019) | **0.822**  **(0.027)** | 0.771  (0.015) | 0.766  (0.021) | **0.821**  **(0.028)** |
| JMedDeBERTa(c) | 0.769  (0.021) | 0.810  (0.010) | 0.767  (0.025) | 0.764  (0.021) | 0.802  (0.010) | 0.766  (0.025) | 0.762  (0.021) | 0.798  (0.011) | 0.765  (0.025) | 0.759  (0.022) | 0.794  (0.011) | 0.765  (0.025) |

Table S16. Case-level Character Segment Score for 17 encoder/decoder models on the public dataset MRNER-disease. Character-level F1, Recall, and Precision were calculated for each clinical document and averaged. Values are presented as mean (SD).

| MRNER-disease  Each Extraction Targets | F1 | Recall | Precision |
| --- | --- | --- | --- |
|  |  |  |  |
| Decoder Models |  |  |  |
| Instruction Tuning |  |  |  |
| GPTNeox-3.6B | 0.326  (0.042) | 0.308  (0.038) | 0.440  (0.102) |
| LLM-jp-13B v1.0 | 0.566  (0.092) | 0.540  (0.103) | 0.685  (0.099) |
| Swallow-7B | 0.580  (0.060) | 0.607  (0.091) | 0.628  (0.044) |
| Swallow-13B | 0.645  (0.065) | 0.644  (0.112) | 0.732  (0.078) |
| Swallow-70B | 0.647  (0.056) | 0.645  (0.085) | 0.751  (0.078) |
| Swallow-MS-7B | 0.577  (0.044) | 0.584  (0.089) | 0.676  (0.047) |
| Token Classification |  |  |  |
| GPTNeox-3.6B | 0.642  (0.038) | 0.638  (0.069) | 0.716  (0.043) |
| LLM-jp-13B v1.0 | 0.678  (0.035) | 0.669  (0.068) | 0.741  (0.036) |
| Swallow-7B | 0.668  (0.031) | 0.662  (0.072) | 0.725  (0.065) |
| Swallow-13B | 0.655  (0.022) | 0.639  (0.070) | 0.737  (0.034) |
| Swallow-70B | 0.636  (0.032) | 0.629  (0.063) | 0.695  (0.027) |
| Swallow-MS-7B | 0.619  (0.020) | 0.619  (0.036) | 0.670  (0.039) |
| Encoder Models |  |  |  |
| Token Classification |  |  |  |
| DeBERTaV2-base | **0.793**  **(0.020)** | **0.808**  **(0.028)** | 0.812  (0.050) |
| RoBERTa-base | 0.766  (0.017) | 0.775  (0.033) | 0.784  (0.018) |
| JMedRoBERTa | 0.737  (0.014) | 0.727  (0.030) | 0.793  (0.037) |
| JMedDeBERTa(s) | 0.776  (0.022) | 0.751  (0.007) | **0.848**  **(0.025)** |
| JMedDeBERTa(c) | 0.780  (0.019) | 0.791  (0.016) | 0.807  (0.024) |

Table S17. Case-level Token Segment Score for 17 encoder/decoder models on the public dataset MRNER-disease. Token-level F1, Recall, and Precision were calculated for each clinical document and averaged. Values are presented as mean (SD).

| MRNER-disease  Each Extraction Targets | F1 | Recall | Precision |
| --- | --- | --- | --- |
|  |  |  |  |
| Decoder Models |  |  |  |
| Instruction Tuning |  |  |  |
| GPTNeox-3.6B | 0.334  (0.041) | 0.313  (0.027) | 0.448  (0.108) |
| LLM-jp-13B v1.0 | 0.578  (0.095) | 0.556  (0.107) | 0.688  (0.099) |
| Swallow-7B | 0.584  (0.062) | 0.613  (0.091) | 0.625  (0.044) |
| Swallow-13B | 0.655  (0.062) | 0.658  (0.108) | 0.734  (0.078) |
| Swallow-70B | 0.651  (0.053) | 0.649  (0.077) | 0.753  (0.077) |
| Swallow-MS-7B | 0.585  (0.050) | 0.590  (0.094) | 0.680  (0.052) |
| Token Classification |  |  |  |
| GPTNeox-3.6B | 0.656  (0.038) | 0.657  (0.065) | 0.719  (0.040) |
| LLM-jp-13B v1.0 | 0.684  (0.036) | 0.680  (0.070) | 0.741  (0.038) |
| Swallow-7B | 0.669  (0.034) | 0.667  (0.070) | 0.722  (0.069) |
| Swallow-13B | 0.662  (0.024) | 0.646  (0.067) | 0.740  (0.038) |
| Swallow-70B | 0.634  (0.031) | 0.626  (0.058) | 0.695  (0.033) |
| Swallow-MS-7B | 0.617  (0.022) | 0.626  (0.033) | 0.648  (0.045) |
| Encoder Models |  |  |  |
| Token Classification |  |  |  |
| DeBERTaV2-base | **0.807**  **(0.022)** | **0.823**  **(0.028)** | 0.821  (0.052) |
| RoBERTa-base | 0.782  (0.024) | 0.799  (0.031) | 0.792  (0.026) |
| JMedRoBERTa | 0.730  (0.025) | 0.730  (0.035) | 0.774  (0.034) |
| JMedDeBERTa(s) | 0.750  (0.027) | 0.726  (0.025) | **0.832**  **(0.032)** |
| JMedDeBERTa(c) | 0.796  (0.017) | 0.809  (0.012) | 0.815  (0.024) |

Table S18. Character-level match between the gold testset input sentences and model outputs after removing @@/## markers.

In this study, we constructed a character-level binary mask (1 for the target concept, 0 otherwise) based on the spans indicated by @@...## in the output. We then aggregated TP/FP/FN over this mask to compute precision, recall, and F1. For negative examples (sentences whose gold annotations contain no tagged spans), the gold mask was entirely 0; therefore, as long as the model output contained no @@...## spans, the predicted mask was also entirely 0. In such cases, even if the reproduced sentence exhibited minor discrepancies from the input (e.g., whitespace or punctuation differences), it did not affect the primary evaluation metrics as long as these differences occurred outside @@...##. In contrast, if the model output contained @@...## in a negative example, that span was treated as a predicted positive, increasing FP and lowering the score. Because instruction-following behavior (i.e., reproducing the same sentence when no concepts were present) was orthogonal to the primary evaluation, we additionally measured string-level agreement between the input and the output after removing prompt-derived text and the @@/## markers. The five-fold mean exact-match rate was 98.40% (SD 0.42%). Most mismatches were minor: considering only the mismatched cases, the mean similarity measured by SequenceMatcher was 0.993 (SD 0.002), the mean Levenshtein edit distance was 7.54 (SD 1.99), and the mean length difference was −2.30 (SD 1.82).

| Metric | 5CV (SD) |
| --- | --- |
| Exact Match Rate [%] | 98.399 (0.418) |
| Mismatch Rate [%] | 1.601 (0.418) |
| Mismatch Count | 63.000 (16.462) |
| Mean Similarity (Mismatches Only) | 0.993 (0.002) |
| Mean Edit Distance (Levenshtein, Mismatches Only) | 7.540 (1.992) |
| Mean Length Difference (Mismatches Only) | -2.300 (1.822) |

Table S19. Gold entity length distribution in the test set (character count).

To examine whether length weighting in the weighted soft matching score could mask weak performance on short entities, we analyzed the length distribution of gold entities. The breakdown is as follows: 1–2 chars (9.06%), 3–6 (33.07%), 7–10 (21.96%), 11–20 (18.39%), and ≥21 (17.51%). Entities of ≤10 characters account for 64.09% of all targets.

| length | count | ratio |
| --- | --- | --- |
| 1–2 chars | 22890 | 9.06% |
| 3–6 chars | 83529 | 33.07% |
| 7–10 chars | 55478 | 21.96% |
| 11–20 chars | 46460 | 18.39% |
| ≥21 chars | 44222 | 17.51% |

Table S20. Length-stratified precision, recall, and F1 under the weighted soft matching score. Values are presented as mean (SD).

We conducted a length-stratified evaluation to ensure that performance on short but clinically critical entities is not obscured. Gold entities were grouped into five character-length bins (1–2, 3–6, 7–10, 11–20, and ≥21), and precision, recall, and F1 were computed. Under the weighted soft-matching score at p=1, Swallow-70B achieved an F1 of 0.814 (SD 0.017) for 1–2 characters and remained strong for 3–10 characters (F1=0.738 (SD 0.018) – 0.736 (SD 0.024)), whereas performance decreased for ≥21 characters (F1=0.677 (SD 0.015)). In contrast, the encoder model (JMedDeBERTa(s)) exhibited lower F1 on extremely short spans (1–2 characters), which comprise only 9% of all entities. However, for spans of three or more characters, it consistently outperformed the decoder model across bins, and its F1 did not substantially degrade even for long spans (≥10 characters).

| J-CaseMap  All Extraction Targets | length | p=1 | | | p=1.5 | | | p=2 | | | p=100 | | |
| --- | --- | --- | --- | --- | --- | --- | --- | --- | --- | --- | --- | --- | --- |
|  |  | F1 | Recall | Precision | F1 | Recall | Precision | F1 | Recall | Precision | F1 | Recall | Precision |
| Decoder Models |  |  |  |  |  |  |  |  |  |  |  |  |  |
| Instruction Tuning |  |  |  |  |  |  |  |  |  |  |  |  |  |
| Swallow-70B | 1–2 chars | 0.814  (0.017) | 0.822  (0.034) | 0.806  (0.004) | 0.814  (0.017) | 0.822  (0.034) | 0.806  (0.004) | 0.814  (0.017) | 0.822  (0.034) | 0.806  (0.004) | 0.814  (0.017) | 0.822  (0.034) | 0.806  (0.004) |
|  | 3–6 chars | 0.738  (0.018) | 0.730  (0.044) | 0.749  (0.012) | 0.738  (0.018) | 0.729  (0.044) | 0.749  (0.012) | 0.738  (0.018) | 0.729  (0.044) | 0.749  (0.012) | 0.738  (0.018) | 0.728  (0.044) | 0.749  (0.012) |
|  | 7–10 chars | 0.736  (0.024) | 0.721  (0.047) | 0.753  (0.007) | 0.735  (0.024) | 0.719  (0.047) | 0.753  (0.007) | 0.734  (0.024) | 0.718  (0.047) | 0.752  (0.007) | 0.733  (0.024) | 0.717  (0.047) | 0.752  (0.007) |
|  | 11–20 chars | 0.758  (0.019) | 0.742  (0.038) | 0.774  (0.006) | 0.755  (0.019) | 0.739  (0.038) | 0.773  (0.006) | 0.755  (0.019) | 0.738  (0.038) | 0.773  (0.006) | 0.753  (0.019) | 0.736  (0.038) | 0.772  (0.006) |
|  | ≥21 chars | 0.677  (0.015) | 0.627  (0.033) | 0.738  (0.037) | 0.650  (0.014) | 0.600  (0.031) | 0.712  (0.043) | 0.641  (0.014) | 0.591  (0.030) | 0.704  (0.045) | 0.630  (0.014) | 0.578  (0.030) | 0.694  (0.046) |
| Encoder Models |  |  |  |  |  |  |  |  |  |  |  |  |  |
| Token Classification |  |  |  |  |  |  |  |  |  |  |  |  |  |
| JmedDeBERTa(s) | 1–2 chars | 0.695  (0.010) | 0.932  (0.015) | 0.554  (0.012) | 0.695  (0.010) | 0.932  (0.015) | 0.554  (0.012) | 0.695  (0.010) | 0.932  (0.015) | 0.554  (0.012) | 0.695  (0.010) | 0.932  (0.015) | 0.554  (0.012) |
|  | 3–6 chars | 0.741  (0.003) | 0.868  (0.014) | 0.647  (0.010) | 0.741  (0.003) | 0.867  (0.014) | 0.647  (0.010) | 0.741  (0.003) | 0.867  (0.014) | 0.647  (0.010) | 0.740  (0.003) | 0.866  (0.014) | 0.647  (0.010) |
|  | 7–10 chars | 0.764  (0.006) | 0.838  (0.009) | 0.702  (0.012) | 0.761  (0.006) | 0.832  (0.009) | 0.701  (0.012) | 0.760  (0.006) | 0.829  (0.009) | 0.701  (0.012) | 0.758  (0.006) | 0.826  (0.010) | 0.701  (0.012) |
|  | 11–20 chars | 0.796  (0.004) | 0.834  (0.007) | 0.762  (0.010) | 0.791  (0.004) | 0.823  (0.007) | 0.762  (0.010) | 0.789  (0.004) | 0.819  (0.007) | 0.761  (0.010) | 0.786  (0.005) | 0.813  (0.007) | 0.761  (0.010) |
|  | ≥21 chars | 0.756  (0.005) | 0.700  (0.015) | 0.823  (0.012) | 0.719  (0.006) | 0.643  (0.016) | 0.815  (0.012) | 0.706  (0.006) | 0.625  (0.016) | 0.812  (0.013) | 0.690  (0.007) | 0.603  (0.017) | 0.808  (0.013) |

Table S21. Length-stratified precision, recall, and F1 under the marker matching score (non-length-weighted). Values are presented as mean (SD).

We performed a length-stratified evaluation to ensure that performance on short but clinically critical entities is not obscured. Gold entities were grouped into five character-length bins (1–2, 3–6, 7–10, 11–20, and ≥21), and precision, recall, and F1 were computed. The marker matching score shows consistent behavior for short spans (e.g., 1–2 characters: F1=0.658 (SD 0.011), recall=0.933 (SD 0.015), precision=0.508 (SD 0.012)).

| J-CaseMap  All Extraction Targets | length | p=1 | | | p=1.5 | | | p=2 | | | p=100 | | |
| --- | --- | --- | --- | --- | --- | --- | --- | --- | --- | --- | --- | --- | --- |
|  |  | F1 | Recall | Precision | F1 | Recall | Precision | F1 | Recall | Precision | F1 | Recall | Precision |
| Decoder Models |  |  |  |  |  |  |  |  |  |  |  |  |  |
| Instruction Tuning |  |  |  |  |  |  |  |  |  |  |  |  |  |
| Swallow-70B | 1–2 chars | 0.814  (0.017) | 0.823  (0.034) | 0.806  (0.004) | 0.814  (0.017) | 0.823  (0.034) | 0.806  (0.004) | 0.814  (0.017) | 0.823  (0.034) | 0.806  (0.004) | 0.814  (0.017) | 0.823  (0.034) | 0.806  (0.004) |
|  | 3–6 chars | 0.734  (0.017) | 0.725  (0.043) | 0.745  (0.013) | 0.734  (0.017) | 0.725  (0.043) | 0.745  (0.013) | 0.734  (0.017) | 0.725  (0.043) | 0.745  (0.013) | 0.734  (0.017) | 0.724  (0.043) | 0.745  (0.013) |
|  | 7–10 chars | 0.737  (0.023) | 0.722  (0.046) | 0.753  (0.007) | 0.735  (0.023) | 0.720  (0.046) | 0.753  (0.007) | 0.735  (0.023) | 0.719  (0.046) | 0.753  (0.007) | 0.734  (0.023) | 0.718  (0.046) | 0.753  (0.007) |
|  | 11–20 chars | 0.757  (0.019) | 0.743  (0.039) | 0.773  (0.005) | 0.755  (0.019) | 0.740  (0.038) | 0.772  (0.005) | 0.755  (0.019) | 0.739  (0.038) | 0.771  (0.005) | 0.753  (0.019) | 0.738  (0.038) | 0.771  (0.005) |
|  | ≥21 chars | 0.727  (0.016) | 0.679  (0.035) | 0.783  (0.016) | 0.713  (0.016) | 0.663  (0.034) | 0.773  (0.017) | 0.708  (0.015) | 0.657  (0.033) | 0.769  (0.018) | 0.701  (0.015) | 0.648  (0.033) | 0.765  (0.018) |
| Encoder Models |  |  |  |  |  |  |  |  |  |  |  |  |  |
| Token Classification |  |  |  |  |  |  |  |  |  |  |  |  |  |
| JmedDeBERTa(s) | 1–2 chars | 0.658  (0.011) | 0.933  (0.015) | 0.508  (0.012) | 0.658  (0.011) | 0.933  (0.015) | 0.508  (0.012) | 0.658  (0.011) | 0.933  (0.015) | 0.508  (0.012) | 0.658  (0.011) | 0.933  (0.015) | 0.508  (0.012) |
|  | 3–6 chars | 0.735  (0.003) | 0.869  (0.013) | 0.637  (0.011) | 0.734  (0.003) | 0.868  (0.013) | 0.637  (0.011) | 0.734  (0.003) | 0.867  (0.013) | 0.637  (0.011) | 0.734  (0.003) | 0.867  (0.013) | 0.637  (0.011) |
|  | 7–10 chars | 0.763  (0.005) | 0.839  (0.009) | 0.700  (0.012) | 0.761  (0.005) | 0.833  (0.010) | 0.700  (0.012) | 0.760  (0.005) | 0.831  (0.010) | 0.700  (0.012) | 0.758  (0.005) | 0.828  (0.010) | 0.699  (0.012) |
|  | 11–20 chars | 0.796  (0.005) | 0.836  (0.007) | 0.759  (0.011) | 0.791  (0.005) | 0.826  (0.007) | 0.758  (0.011) | 0.789  (0.005) | 0.822  (0.007) | 0.758  (0.011) | 0.786  (0.005) | 0.816  (0.007) | 0.758  (0.011) |
|  | ≥21 chars | 0.787  (0.002) | 0.757  (0.013) | 0.819  (0.012) | 0.764  (0.003) | 0.721  (0.013) | 0.813  (0.012) | 0.756  (0.003) | 0.709  (0.014) | 0.811  (0.012) | 0.746  (0.004) | 0.692  (0.014) | 0.809  (0.013) |

Figure S3. Example of an "error" by JMedDeBERTa(s) where the prediction differs partially from the reference label but is considered clinically acceptable (F1 = 0.755). Blue highlights indicate the Gold Standard for clinically essential expressions, and red highlights indicate the model prediction for the Japanese case report (and its English translation). Although the model selects spans with different boundaries or granularity than the reference labels, causing a drop in F1 score, many predicted phrases remain clinically meaningful, suggesting that some discrepancies reflect annotation subjectivity rather than distinct model errors.


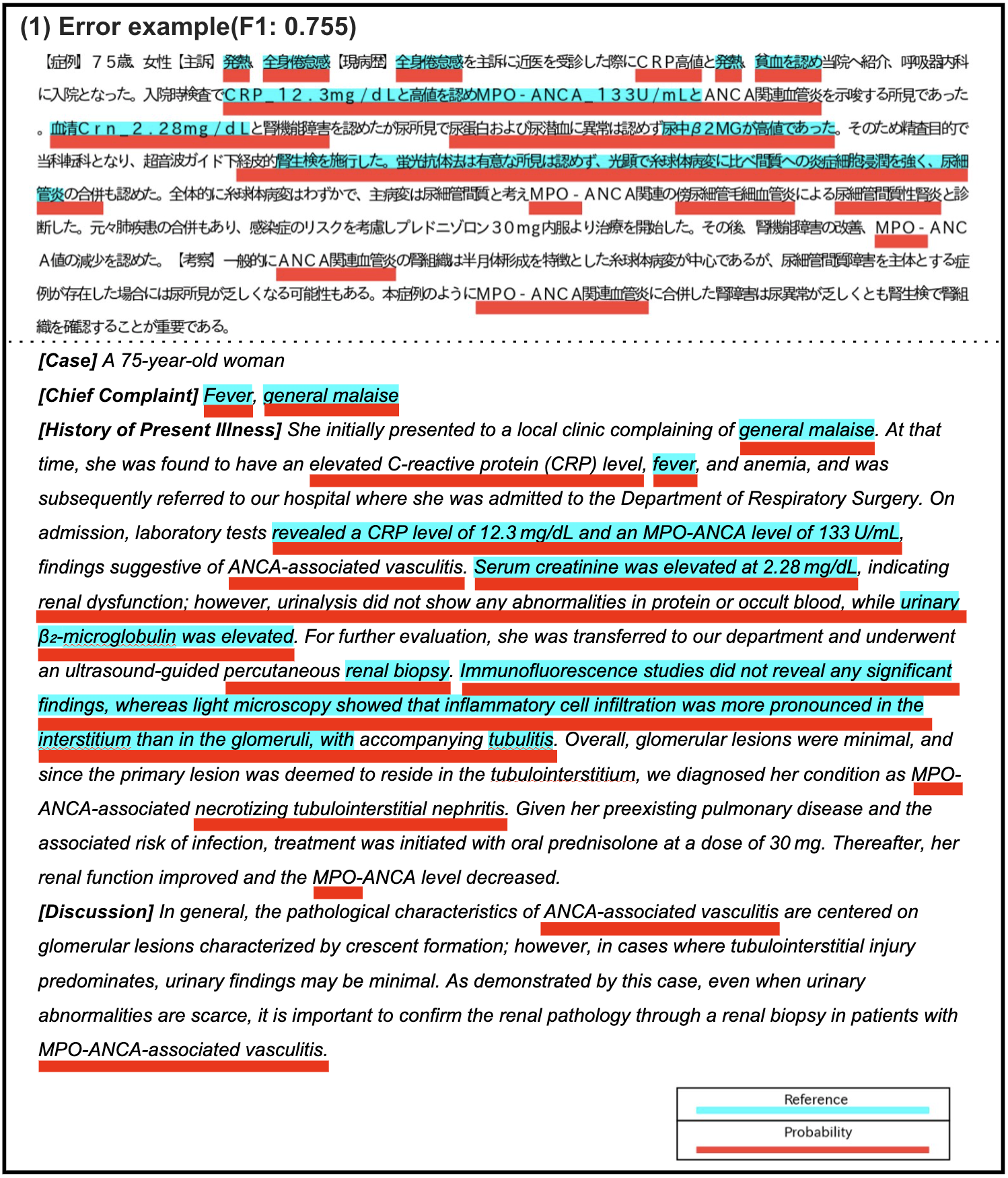


Figure S4. Second "error" example in JMedDeBERTa(s) (F1 = 0.752). Similar to Figure S3, blue indicates reference annotations and red indicates model predictions for the Japanese case report and its English translation. Some predicted spans extend beyond or deviate from the reference labels, lowering the F1 score, but the additionally extracted phrases contain relevant diagnostic information. This indicates limitations in the gold standard itself for evaluating long expressions.


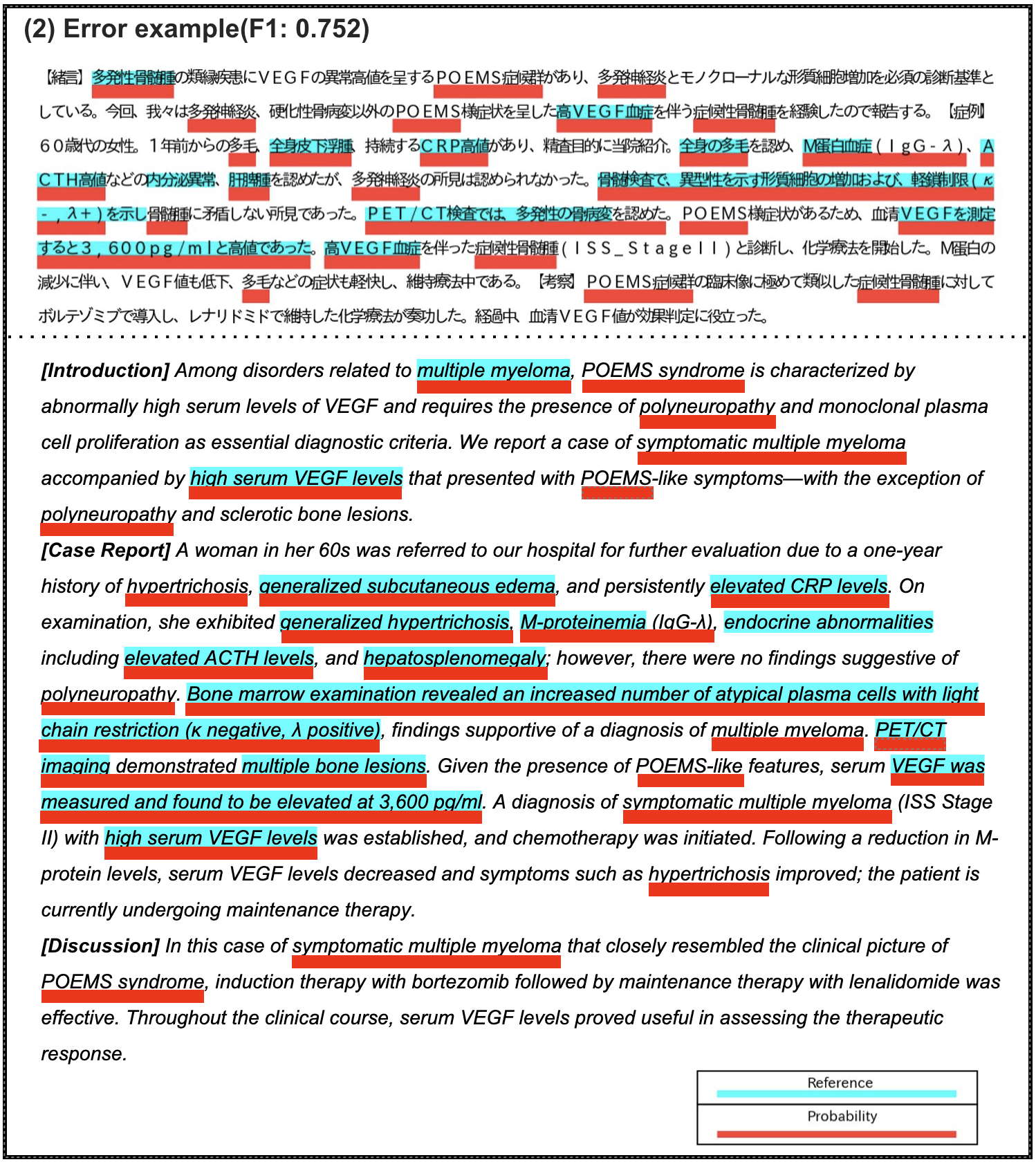


Figure S5. Third example showing discrepancy between JMedDeBERTa(s) and reference labels (F1 = 0.739). Blue highlights indicate the Gold Standard for clinically essential expressions, and red highlights indicate model predictions. Not all discrepancies imply clinically incorrect extractions, suggesting that the reported scores may underestimate the actual model performance.


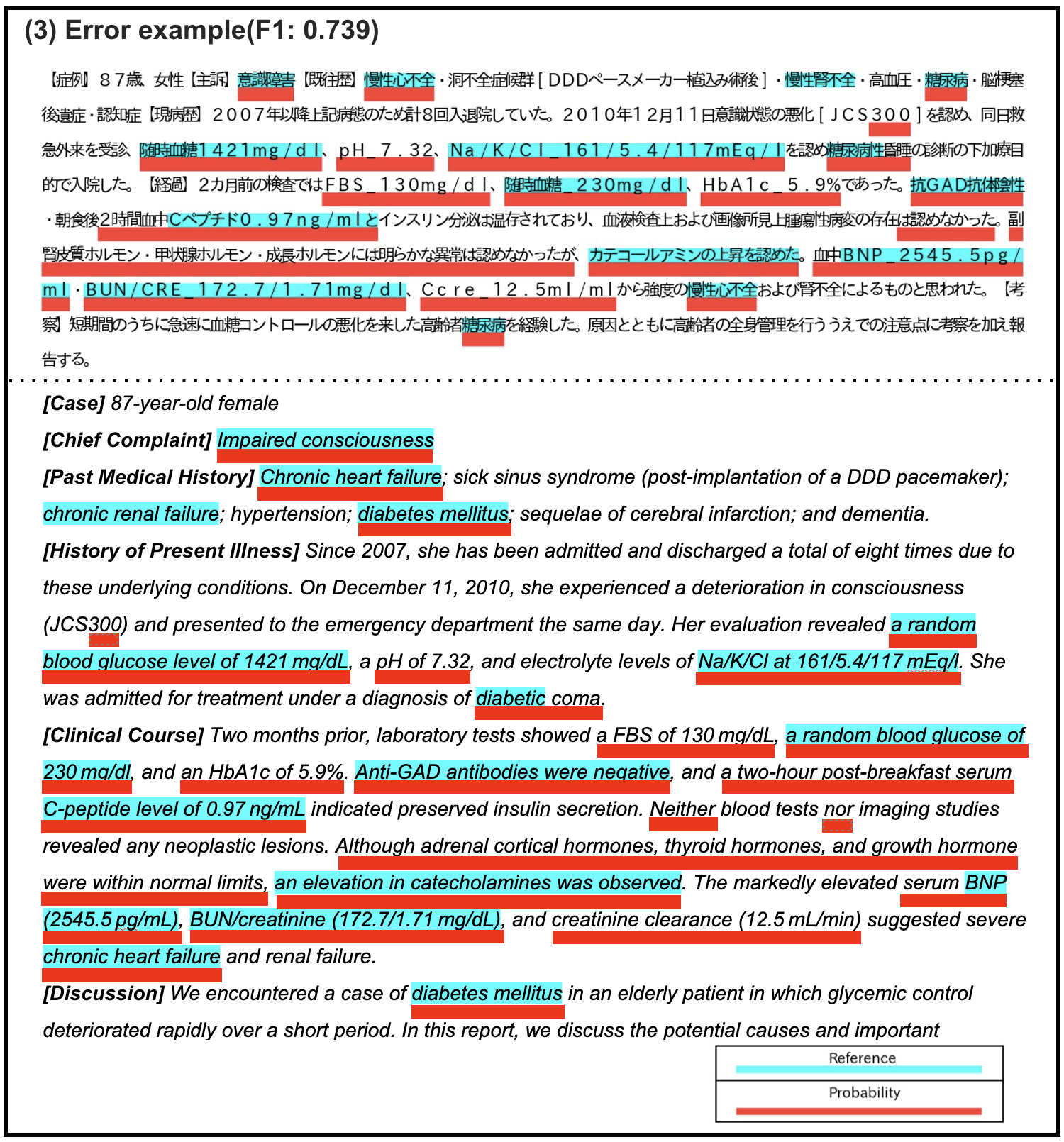

Supplement: Multimedia Appendix 1 [file jmir-v28-e78681-s001.docx]
